# Genetic evidence and cross-species functional characterization implicate *CNN2* in age-related macular degeneration susceptibility

**DOI:** 10.1073/pnas.2533682123

**Published:** 2026-09-02

**Authors:** Fei-Fei Cheng, Hao Mou, Zhenyu Liu, Chang-Jun Zhang, You-Yuan Zhuang, Zhen Wu, Xin-Ran Wen, Angli Xue, Xiao Zhang, Jian Yang, Zi-Bing Jin

**Affiliations:** ^a^ New Cornerstone Science Laboratory, School of Life Sciences, Westlake University, Hangzhou, Zhejiang 310030, China; ^b^ Beijing Institute of Ophthalmology, Beijing Tongren Hospital, Capital Medical University, Beijing Key Laboratory for Ophthalmic Cell and Gene Therapy, Beijing 100730, China; ^c^ Division of Ophthalmic Genetics, The Eye Hospital, Wenzhou Medical University, Wenzhou 325027, China; ^d^ Institute for Molecular Bioscience, The University of Queensland, Brisbane, QLD 4072, Australia; ^e^ Translational Genomics Program, Garvan Institute of Medical Research, Sydney, NSW 2010, Australia; ^f^ Faculty of Medicine and Health, University of New South Wales, Sydney, NSW 2052, Australia; ^g^Westlake Laboratory of Life Sciences and Biomedicine, Hangzhou, Zhejiang 310030, China; ^h^Chinese Institutes for Medical Research, Beijing 100069, China

**Keywords:** age-related macular degeneration, CNN2, statistical genetics, photoreceptor degeneration, retinal neurovascular homeostasis

## Abstract

Age-related macular degeneration (AMD) is a leading cause of irreversible visual impairment in the aging population globally. Although genome-wide association studies (GWAS) have identified many AMD susceptibility loci, the genes and mechanisms underlying many of these associations remain unresolved. Here, we integrated expression quantitative trait locus (eQTL) data with AMD GWAS to prioritize nine putative genes. Through in vivo screening in zebrafish, we demonstrated that the downregulation of *cnn2* and *sarm1* expression led to ocular structural abnormalities and visual functional impairment. Subsequent mouse model studies confirmed that *Cnn2* deficiency affected photoreceptor structure and function, impaired contrast sensitivity, and caused abnormalities in cone cell immunostaining. Given that *CNN2* is predominantly expressed in endothelial cells, we propose that endothelial dysfunction may cascade to impair photoreceptor function. Collectively, through in silico prioritization and cross-species functional characterization, we identify *CNN2* as a candidate susceptibility gene in AMD pathogenesis, providing vital underlying mechanistic insights.

## Significance

Age-related macular degeneration (AMD) is the most common cause of irreversible vision loss among older adults and is projected to affect approximately 288 million people worldwide by 2040. Identifying the genes and cellular mechanisms underlying established AMD risk loci is essential for understanding disease pathogenesis and facilitating therapeutic development. By integrating human genetic analyses with functional studies in zebrafish and mouse models, we identify *CNN2* as a compelling candidate AMD gene and demonstrate that *CNN2* deficiency disrupts photoreceptor structure and visual function. Our findings support a model in which *CNN2* deficiency perturbs retinal neurovascular homeostasis, potentially contributing to photoreceptor dysfunction in AMD.

Age-related macular degeneration (AMD) is a chronic retinal disease that causes irreversible central vision loss among older adults, characterized by the dysfunction of retinal pigment epithelium (RPE) and progressive loss of photoreceptors in the macula (1, 2). The disorder leads to a decline in central vision, causing limitations in everyday activities such as reading, walking, and face recognition. Globally, the prevalence of AMD in the 45 to 85 age group stands at 8.69%, with projections estimating its impact on 288 million people worldwide by 2040 (3). As such, AMD poses a substantial health and socioeconomic burden for individuals and communities, particularly in aging populations (4).

AMD is one of the most genetically well-defined complex eye diseases because highly replicable genetic loci have been identified, explaining a remarkably high percentage of the disease heritability. GWAS with increasing sample sizes have identified at least 63 susceptibility loci, collectively accounting for over 50% of the heritability of susceptibility (5–8). These discoveries have provided valuable insights into the genetic architecture of AMD (8–10) and have facilitated the prioritization of candidate genes. However, for many associated loci, the susceptibility genes and biological mechanisms remain unresolved, and most nominated candidate genes have not been functionally evaluated in animal models. Considerable progress has been made in understanding the impact of specific genetic variants associated with AMD (11–13). For instance, a zebrafish model has shed light on the impact of a rare AMD-associated variant in the *CFI* gene on hyaloid vessels in the retina (11). Additionally, detectable visual dysfunction and retinal abnormalities have been observed in a 2-y-old *Cfh*-knockout (*Cfh*^−/−^) mouse model (12), representing one of the most significant susceptibility loci found in humans. Nevertheless, considering the complex and multifactorial nature of AMD, it is crucial to identify and characterize the genes underlying the remaining unsolved genetic associations. An efficient and cost-effective strategy is needed to screen candidate genes and evaluate their functional relevance to AMD pathogenesis.

**Fig. 1.**
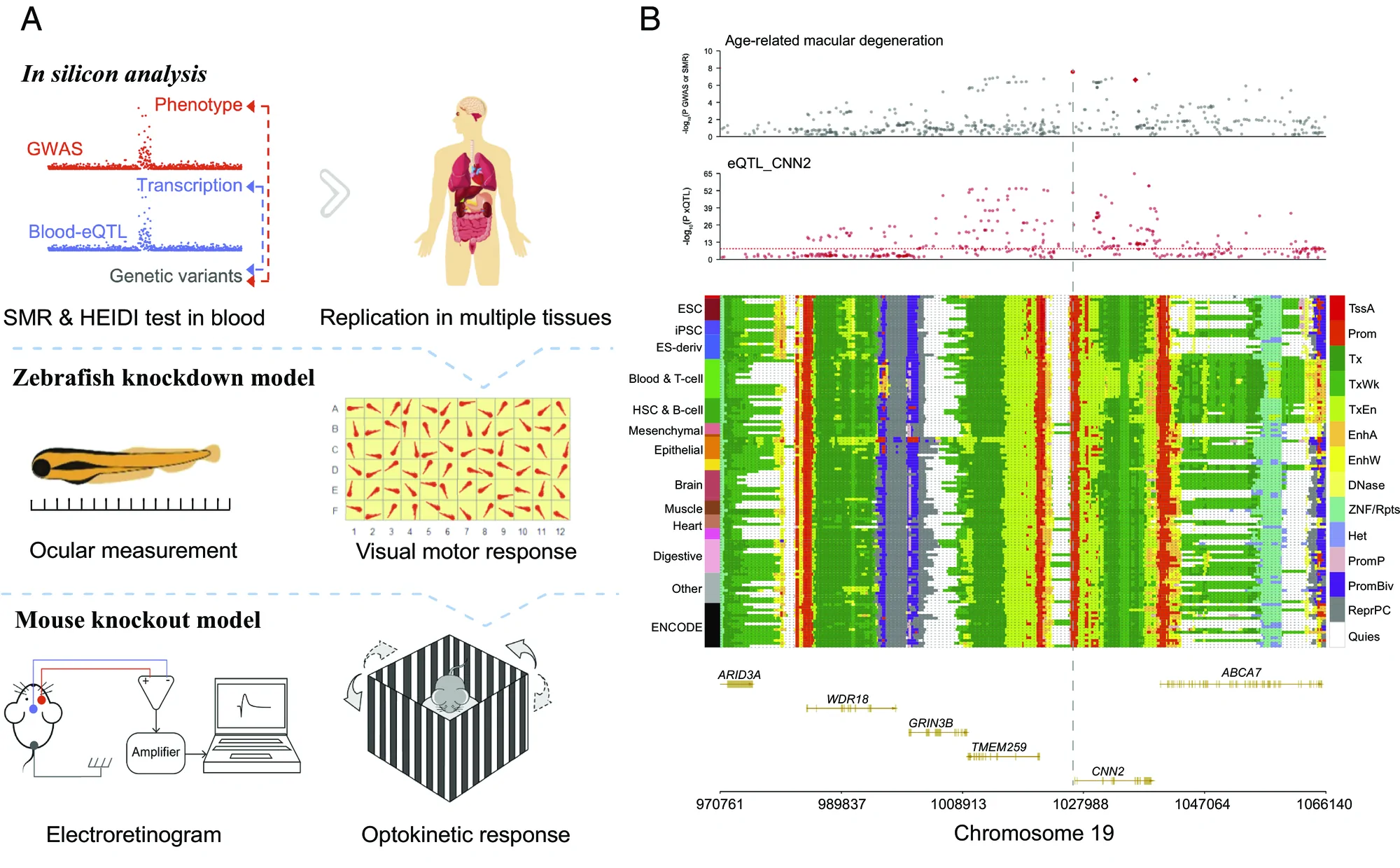
An integrative strategy identifying putative causal genes in AMD. (*A*) Schematic of the study design. First, blood eQTL and AMD GWAS data were integrated through SMR and HEIDI to identify expression–phenotype associations prior to replication in retina or 48 other human tissues from the GTEx dataset. Second, functional experiments were conducted using knockdown zebrafish models to assess ocular sizes and light-induced visual behavioral patterns. Finally, knockout mouse models were employed to evaluate visual perception as assessed by electroretinogram (ERG) measurements and optokinetic responses (OKRs). (*B*) Prioritization of candidate genes and regulatory elements at the *CNN2* locus for AMD. The *Top* panel displays the association signals (−log_10_*P*) from the AMD GWAS. The *Middle* panel illustrates the eQTL association (−log_10_*P*) between these variants and *CNN2* expression. The *Bottom* panel presents chromatin state annotations across 127 primary cells and tissue types from the Roadmap Epigenomics Mapping Consortium. The alignment of GWAS and eQTL peaks with active regulatory states prioritizes *CNN2* as a susceptibility gene for AMD.

In this study, we developed an integrative strategy combining in silico analysis, functional screening in zebrafish, and in vivo characterization in mouse models (Fig. 1*A*). Using summary statistics from a large-scale GWAS (6) and eQTL datasets (14), we aimed to test the hypothesis that genetic variants at some of the susceptibility loci affect the risk of AMD through *cis*-regulation of transcription levels. To this end, we employed the summary-data-based Mendelian randomization (SMR) (15), which leverages GWAS and eQTL data from two independent studies, providing enhanced statistical power. We subsequently applied heterogeneity in dependent instruments (HEIDI) approaches to distinguish causality/pleiotropy [i.e., the same causal variant(s) affecting AMD susceptibility and the expression level of a gene] from linkage [i.e., two distinct causal variants in LD, one affecting AMD susceptibility and the other affecting gene expression].

We subsequently used morpholino oligonucleotide (MO)-induced knockdown in zebrafish as an initial in vivo screening platform to evaluate the candidate susceptibility genes identified through the in silico analysis. Established morphological and functional assays were used to assess ocular development and visual phenotypes. Candidate genes supported by the zebrafish screen were then investigated in mouse models, which enable more detailed evaluation of mammalian retinal structure and visual function, to assess their relevance to AMD-related retinal phenotypes. In parallel, we analyzed retinal transcriptomic datasets to explore the cellular and molecular mechanisms underlying the observed phenotypes. Overall, the research workflow, combining integrative genetic analyses with experimental characterization in zebrafish and mouse models, represents an effective and efficient paradigm for identifying candidate susceptibility genes implicated in complex human diseases.

## Results

### Association of Gene Expression With AMD Risk Through Integrative Analysis.

We used SMR (15) to identify genes exhibiting associations between expression levels and AMD risk using GWAS and eQTL summary data. The GWAS summary data were obtained from the International AMD Genomics Consortium with 16,144 AMD cases and 17,832 controls (6). The eQTL summary data were generated by the Consortium for the Architecture of Gene Expression (CAGE) from a study involving 2,765 individuals, with 36,778 gene expression probes in peripheral blood samples (14) [Note: At the time of the original analysis, this dataset held the largest sample size for GWAS and eQTL data. Reanalysis using recent data (8) produced consistent findings (*SI Appendix*, section 1 and Dataset S1)].

In total, we identified 16 genes, tagged by 21 probes, which reached genome-wide significance ($P_{\text{SMR}}=5.9\times 10^{-6}$, Bonferroni corrected for 8,459 tests, i.e., 8,459 probes with at least an eQTL at *P*_eQTL_ < $5.0\times 10^{-8}$) (Dataset S1). We then employed the HEIDI method (15) to reject SMR associations due to linkage (removing probes with *P*_HEIDI_ < 0.05) (Methods). The LD information required for the HEIDI test was calculated from genotype data of 20,000 unrelated individuals of European ancestry randomly selected from the UK Biobank (16). Consequently, 9 genes (tagged by 12 probes) were retained with their functional annotation summarized (*SI Appendix*, Table S1), some of which, namely *BLOC1S1, PILRB,* and *TMEM199,* were previously reported using a different strategy to integrate AMD GWAS with retinal eQTL data (17).

### Replication of the SMR Associations in the Retina and Other Tissues.

Recognizing that genetic effects can be tissue-dependent, we validated our blood-based SMR findings by testing the nine significant genes in the retina to confirm their ocular relevance. The retinal eQTL summary data were obtained from 406 postmortem donors (17), available in the Eye Genotype Expression (EyeGex) database (https://gtexportal.org/home/datasets). Four of the nine genes were replicated at $P_{\mathrm{smr}}=5.6\times 10^{-3}$ (i.e., 0.05/9) with consistent effect directions, a relatively high replication rate given the small sample size of the EyeGex data (*SI Appendix*, Fig. S1 and Dataset S1).

It is unclear whether AMD is localized solely in the affected retina or if it represents an ocular manifestation of a broader systemic process, since risk factors such as cigarette smoking, nutrition, and cardiovascular disease have a significant impact on the disease progression (18–22). To address this, we conducted SMR analysis for the nine genes in a wider range of tissues available in the GTEx project (23) (*SI Appendix*, Fig. S1 and Dataset S1). The results showed that *PILRB*, a key activator of immune function, exhibited significance across all 48 human tissues, with effect sizes ranging from 0.081 (in the retina) to 0.249 (in whole blood), implying the involvement of systemic immune pathways in AMD pathogenesis. Supporting this genetic evidence, a previous functional study has shown that *Pilrb* knockout mice had an apparent reduction in electroretinography (ERG) amplitudes from postnatal day 15 through 12 mo of age (13). In fact, all nine genes demonstrated significance in at least two tissues, indicating that their genetic associations with AMD were not tissue-specific.

### Knockdown of *cnn2* or *sarm1* Led to Ocular Abnormalities in Zebrafish.

**Fig. 2.**
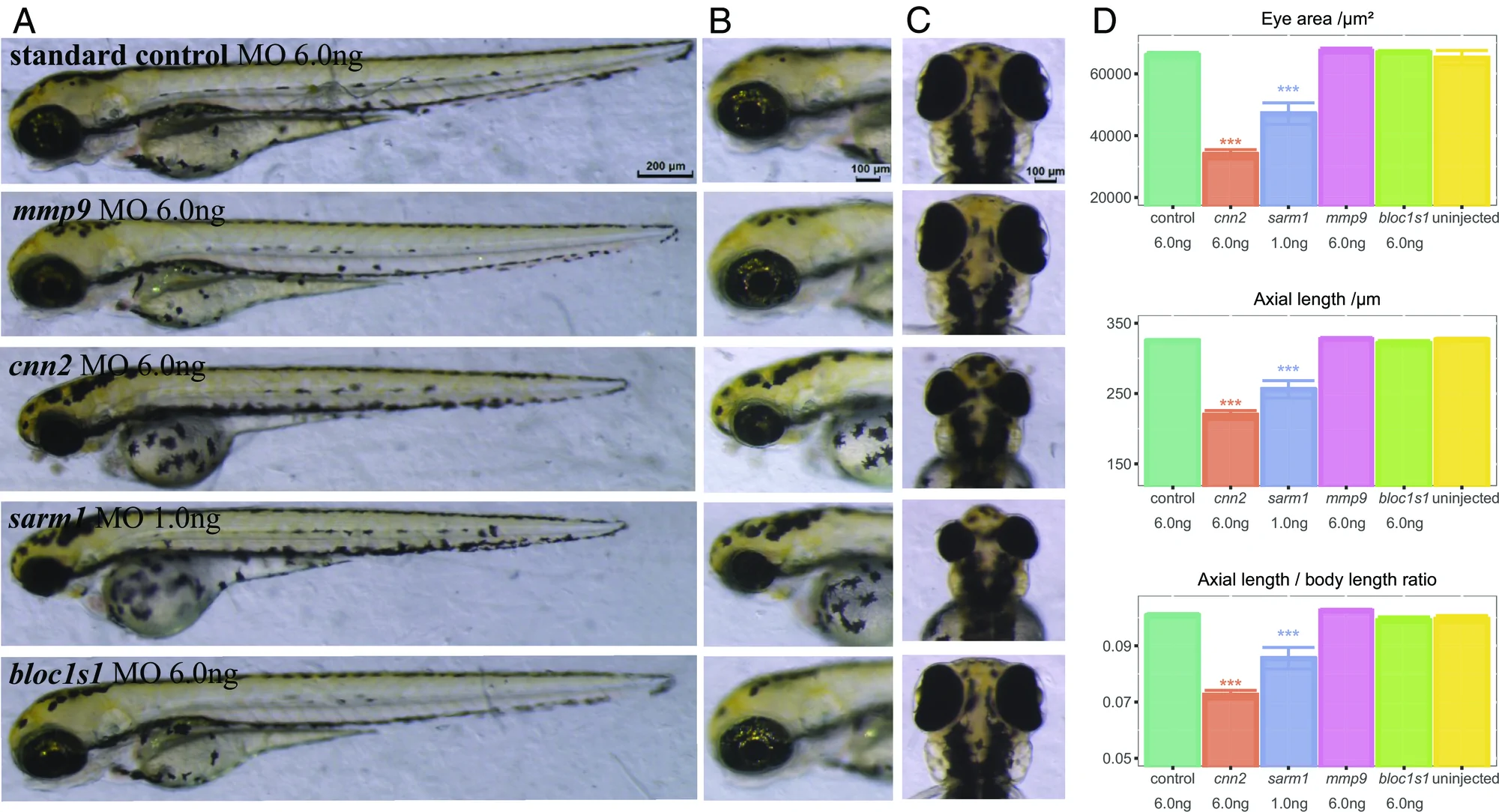
Phenotypes of *cnn2*-, *sarm1*-, *mmp9*-, and *bloc1s1*-deficient zebrafish. (*A*) Lateral view of whole bodies. (*B*) Magnified lateral views of zebrafish eyeballs revealing a noticeable decrease in eye area in *cnn2-* and *sarm1*-deficient fish. (*C*) Vertical views of eyeballs demonstrating a reduction in axial length in *cnn2-* and *sarm1*-knockdown larvae. (*D*) Quantification of the eye area, axial length, and the ratio of axial length to body length. Bar plots represent the mean ± SEM. Two-tailed Student *t* tests were performed to compare each experimental group against the standard control. *P < 0.05, **P < 0.01, ***P < 0.001 for each group. n=10 for each group.

To validate the functional relevance of the prioritized genes in AMD pathogenesis, we sought an animal model capable of gene expression manipulation and reliable assessment of ocular phenotypes. Considering its extensive use in modeling ocular and other disorders (24–26), we chose zebrafish as our primary screening model. Four out of the nine prioritized genes (*CNN2, SARM1, BLOC1S1,* and *MMP9*) were obtained based on having human-zebrafish orthologue similarity above 60% as reported by Ensembl (27) or GeneCards (28) (https://www.ensembl.org; https://www.genecards.org/; *SI Appendix*, Table S2). Subsequently, morpholino (MO) technology was employed to knock down the corresponding genes by blocking mRNA translation. We observed that downregulation of *cnn2* and *sarm1* at doses of 6.0 ng MO and 1.0 ng MO, respectively, led to obvious reductions in axial length (by 32.5% and 21.3%, respectively) and eye area (by 48.5% and 28.5%, respectively) compared to the standard controls at 3 d postfertilization (dpf) (Fig. 2). However, the suppression of *mmp9* and *bloc1s1* did not exhibit significant differences at 3 dpf (Fig. 2 and *SI Appendix*, Fig. S2).

**Fig. 3.**
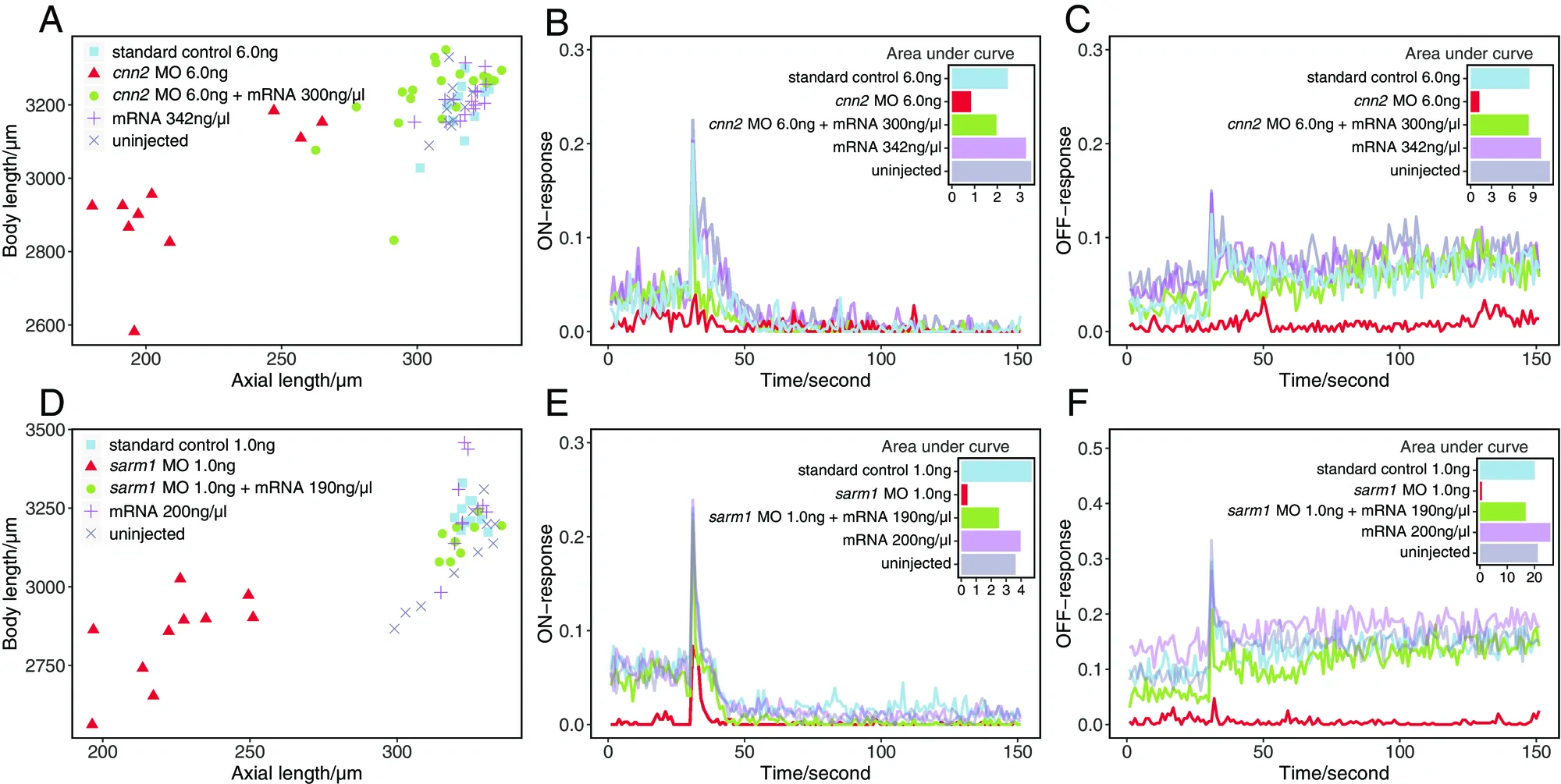
Morphological and functional effects of *cnn2* and *sarm1* knockdown and rescue in zebrafish larvae. (*A*) Scatter plot illustrating the relationship between body length and eyeball axial length in the *cnn2* experiment at 3 d postfertilization (dpf). The *cnn2* MO group showed reduced eye size, which was rescued by *cnn2* mRNA injection. (*B* and *C*) VMR tests evaluating the ON and OFF responses in the *cnn2* experiment at 5 dpf. (*D*) Scatter plot illustrating the relationship between body length and eyeball axial length in the *sarm1* experiment at 3 dpf. The *sarm1* MO group showed reduced eye size, which was rescued by *sarm1* mRNA injection. (*E* and *F*) VMR tests evaluating the ON and OFF responses in the *sarm1* experiment at 5 dpf. Light-on or light-off stimuli occurred at 30 s. Larval motor activity was recorded over time, and the area under the curve represents cumulative motor activity during the 150-s test period. The responses were impaired after *cnn2* or *sarm1* knockdown and restored by the corresponding mRNA injection.

To validate and reinforce our findings, we conducted dose-dependent and rescue experiments. We observed that at a lower dose of *sarm1*-MO (0.5 ng), the *sarm1*-MO morphants exhibited abnormal ocular phenotypes, and these effects intensified with increasing MO dose (from 0.5 to 4.0 ng). This suggests that ocular development may be highly sensitive to the expression level of *sarm1* (*SI Appendix*, Fig. S3). Similarly, the reduction in eye sizes was proportional to the dose of *cnn2*-MO within the range of 4.0 to 6.0 ng (*SI Appendix*, Fig. S4). Importantly, rescue of the affected eyes was achieved by coinjecting mRNA and MO for both genes (Fig. 3 *A* and *D*). The axial length recovered by 95.8% for the *sarm1*-MO and 91.0% for the *cnn2*-MO conditions, respectively.

Additionally, immunostaining was carried out to evaluate the retinal structure (*SI Appendix*, Table S3). Prominent RPE disorganization and cell loss were observed in both *sarm1*-MO and *cnn2*-MO morphants (*SI Appendix*, Fig. S5). Furthermore, reverse transcription-qPCR (RT-qPCR) analysis revealed compromised gene expression of retinal signature genes, including rhodopsin, four types of cone opsins (red-, green-, blue-, and UV-sensitive opsins), and RPE-specific gene rpe65a (*SI Appendix*, Fig. S6). These results provide additional evidence supporting the roles of *CNN2* and *SARM1* in ocular development.

### Knockdown of *cnn2* or *sarm1* Led to Visual Functional Impairment in Zebrafish.

To evaluate the visual condition at the behavioral level, we conducted a visual motor response (VMR) assay for *cnn2-* or *sarm1-*deficient zebrafish morphants. Following a standard protocol (29), zebrafish were individually positioned in the wells of a 96-well plate, and their locomotor responses to light-on and light-off transitions were recorded at 5 dpf. The uninjected control, standard MO control, and *cnn2* mRNA groups exhibited a brief spike in motor activity at approximately 0.20 to 0.22 for the ON response and 0.12 to 0.15 for the OFF response (Fig. 3 *B* and *C*). In contrast, the *cnn2* MO-injected group exhibited an attenuated and delayed response, with the peak reduced by 61.9% for light-on ($P=2.1\times 10^{-3}$) and by 78.6% at 20 s after light-off ($P=4.5\times 10^{-7}$) compared to the standard controls (Fig. 3 *B* and *C*). Notably, coinjection of *cnn2* mRNA with *cnn2* MO restored the visual response to 0.16 for light-on (a 61.5% recovery) and intensified motor activities to 0.08 for light-off (a 45.5% recovery), with an overall improvement in baseline activity (Fig. 3 *B* and *C*). It is important to mention that functional recovery relied on an adequate dose of injected mRNA, as injecting lower amounts of *cnn2* mRNA only achieved partial rescue of visual function (*SI Appendix*, Fig. S7). Similarly, the *sarm1* MO-injected group exhibited impaired visual response, which was rescued by *sarm1* mRNA injection (Fig. 3 *E* and *F*). Taken together, the impaired visually evoked locomotor responses following *cnn2* or *sarm1* knockdown in zebrafish support the involvement of both genes in maintaining normal visual function.

### Knockout of *Cnn2* Gene Caused Visual Functional Impairment in Mice.

We further investigated the roles of *CNN2* and *SARM1* in visual function using mouse models, building on the statistical genetic analysis and the findings from the zebrafish experiments. Mouse models enable a more detailed assessment of mammalian retinal structure and visual function and therefore provide an additional platform for evaluating the phenotypic relevance of the prioritized genes. Because our preliminary results exhibited minimal or no discernible phenotypes in the *Sarm1*-knockout mice at 4.5 mo (*SI Appendix*, section 2 and Figs. S8–S11), we did not pursue extensive phenotypic characterization of this model. In contrast, *CNN2* received additional support from complementary gene-prioritization analyses, including gene-based association analyses [MAGMA (30): $P=2.2\times 10^{-7}$; mBAT-combo (31): $P=3.7\times 10^{-9}$], transcriptome-wide association analysis [TWAS (32): $P=1.9\times 10^{-8}$], and the localization of a fine-mapped genetic variant (33) to the *CNN2* promoter within a RPE/choroid-accessible chromatin region (34) (Fig. 1*B* and *SI Appendix*, section 3 and Fig. S12 and Dataset S2). These convergent lines of genetic evidence provided a rationale for prioritizing the *Cnn2* knockout (*Cnn2*^−/−^) mouse model for detailed investigation. The *Cnn2*-knockout mice were generated using the CRISPR/Cas9 strategy (*SI Appendix*, Fig. S13*A*), and the targeted germline deletion was confirmed by Sanger sequencing (*SI Appendix*, Fig. S13 *B* and *C*). To validate the knockout, we assessed *Cnn2* gene expression levels using quantitative RT-PCR in homozygous (*Cnn2*^−/−^), heterozygous (*Cnn2*^+/−^), and wild-type mice. As expected, *Cnn2* gene expression was absent in the *Cnn2*^−/−^ mice and showed a 54.8% decrease in the heterozygous mice compared to the wild-type mice (*SI Appendix*, Fig. S13*D*). Moreover, immunostaining results further corroborated the absence of Cnn2 protein in the *Cnn2*^−/−^ mice (*SI Appendix*, Fig. S14*A*). In wild-type mice, we found that Cnn2 immunostaining colocalized with CD31, a vascular endothelial cell marker (*SI Appendix*, Fig. S14*B*), indicating that Cnn2 is expressed in retinal vascular endothelial cells.

**Fig. 4.**
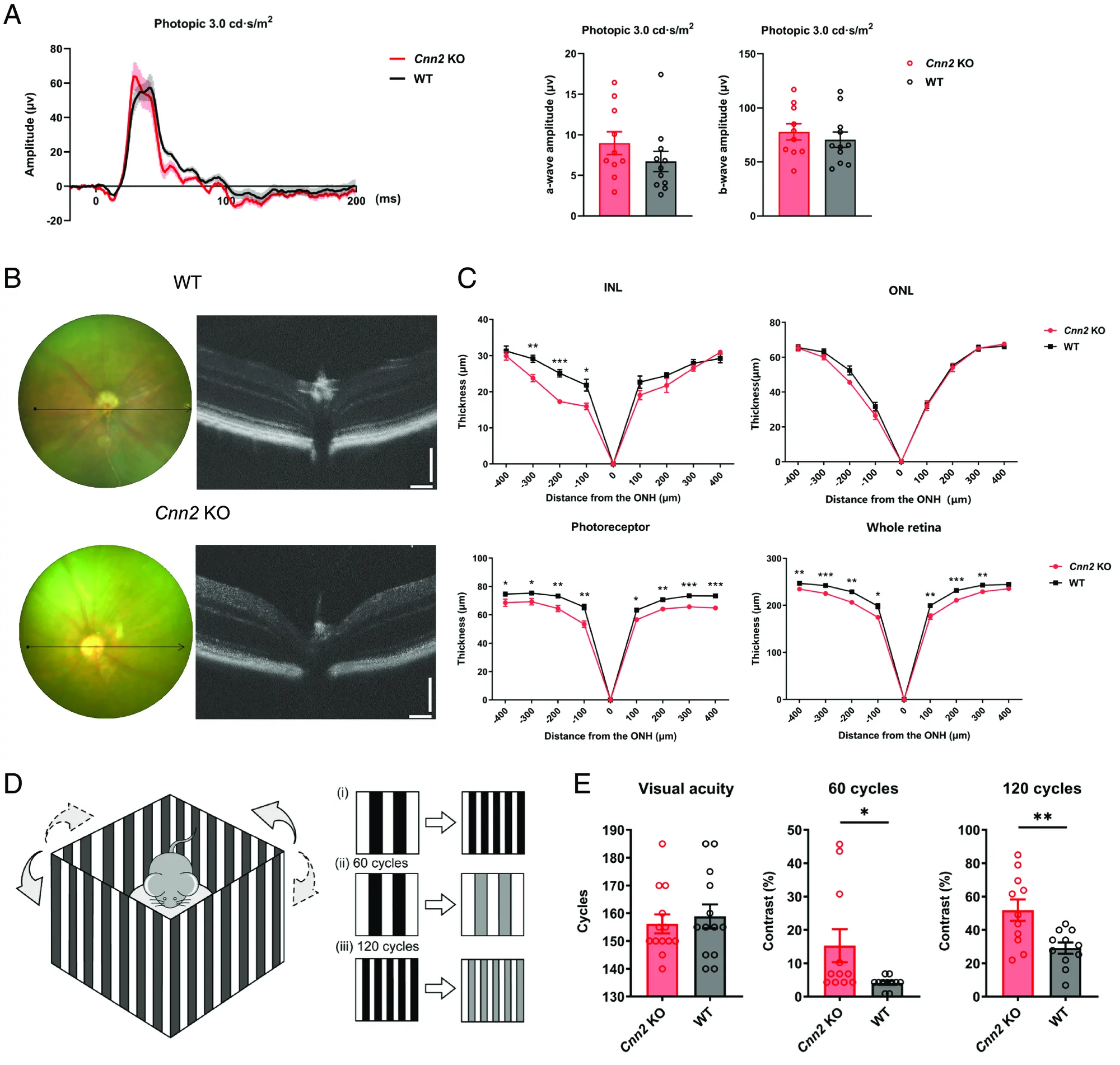
Phenotypes of *Cnn2* knockout mice at 3 mo of age. (*A*) Photopic ERG waveforms and corresponding quantitative measurements of *Cnn2* knockout mice with a sample size of n=11 for wildtype (WT) and n=10 for homozygous (*Cnn2* KO). (*B*) Fundus photography (FP) and Spectral-Domain Optical Coherence Tomography (SD-OCT) images depicting the retinal structure of *Cnn2* knockout mice. The horizontal scale bar represents 100 μm, and vertical scale bar represents 100 μm. (*C*) Quantitative measurements of the SD-OCT results, showcasing the thickness of the inner nuclear layer (INL), outer nuclear layer (ONL), photoreceptor layer and total retina, n (WT) = 7 and n (*Cnn2* KO) = 4. (*D*) Schematic representation of the optokinetic setup used for visual acuity and contrast sensitivity measurements. Both clockwise and counterclockwise rotating screens were used for detection. Visual acuity was assessed based on the density of black and white stripes, while contrast sensitivity was measured at 60 and 120 cycles. (*E*) Results of OKRs, presenting visual acuity and contrast sensitivity measurements under 60 and 120 cycles, respectively. n=13 for visual acuity measurements and n=11 for contrast sensitivity measurements. Bar plots of all panels are shown as the mean ± SEM. Two-tailed Student *t* tests were performed to compare each experimental group against the standard control, * *P* < 0.05, ** *P* < 0.01, *** *P* < 0.001.

To evaluate the functional consequences of *Cnn2* knockout, we assessed the retinal electrical responses using the ERG technique. We did not observe evident abnormalities in the photopic responses of *Cnn2*-knockout mice (Fig. 4*A*). However, our findings revealed a significant compromise in the scotopic response of *Cnn2*-knockout mice at 3 mo of age (*SI Appendix*, Fig. S15). Specifically, at a stimulus intensity of 0.01 cd·s/m^2^, we observed a reduction of 39.2% in a-wave amplitudes (*P* = 0.029), and b-wave amplitudes were reduced by 27.3% compared to the wild-type controls (*P* = 0.015). Preliminary evidence suggested that the impaired scotopic responses in *Cnn2*-knockout mice were detectable as early as 1 mo of age (*SI Appendix*, Figs. S16 *A* and *B* and S17 *A* and *B*). Notably, no significant differences were observed in the scotopic response as the stimulus intensity was increased to 3.0 cd·s/m^2^ or 10.0 cd·s/m^2^, suggesting a modest reduction in retinal sensitivity under low-light conditions.

The OKR was employed to noninvasively assess visual acuity and contrast sensitivity (35). At 3 mo of age, there was no significant difference in visual acuity between the *Cnn2*-knockout and the wild-type mice. Nevertheless, when measuring contrast sensitivity, the *Cnn2*^−/−^ mice exhibited a higher contrast threshold for eliciting head movements, indicating reduced contrast sensitivity. Specifically, the *Cnn2*^−/−^ mice responded to an average contrast of 15.3 ± 4.7% at a spatial frequency of 60 cycles, while the wild-type mice could perceive 4.1 ± 0.5% contrast (*P* = 0.037). As expected, finer contrast settings required higher contrast levels to be perceived. At a spatial frequency of 120 cycles, contrast thresholds of 51.8 ± 6.2% for the *Cnn2*^−/−^ mice and 29.1 ± 3.2% for the wild-type mice were observed (*P* = 0.004, Fig. 4 *D* and *E*). The observation aligns with the clinical manifestation of early AMD, in which contrast sensitivity, particularly for finer contrasts, tends to decline before noticeable changes in visual acuity (36, 37).

### *Cnn2* Knockout Resulted in Degeneration of Photoreceptors in Mice.

**Fig. 5.**
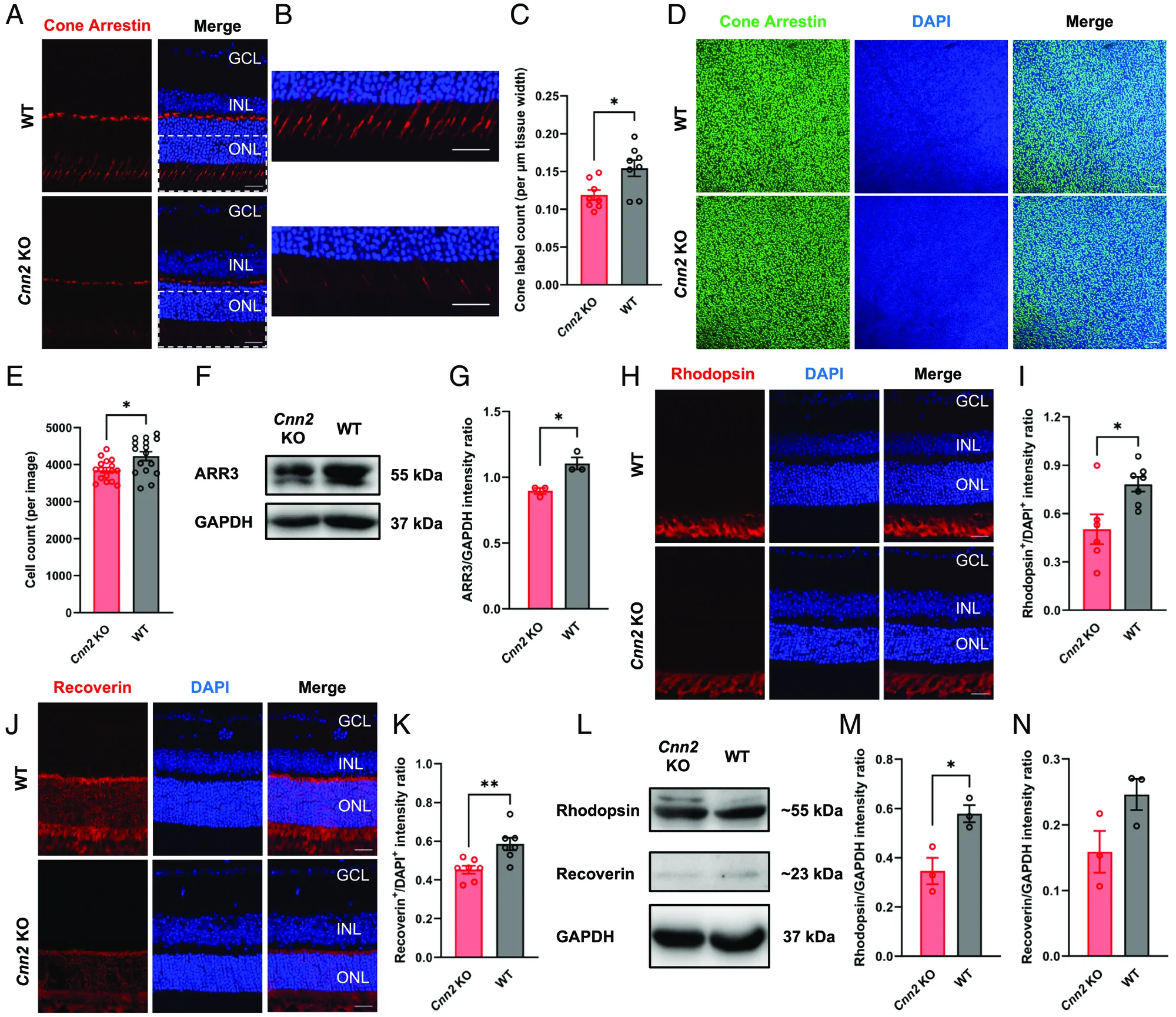
Decreased expression of photoreceptor markers in *Cnn2* KO mice. (*A*) Confocal images showing the expression levels of the cone arrestin marker. (*B*) Enlarged views of the outer segment (OS) region, corresponding to the white dashed boxes in panel (*A*). These images show cone arrestin labeling in frozen section immunostaining. Images were captured at 40× magnification. (Scale bar, 20 μm.) (*C*) Quantification of cone arrestin-labeled cell density in panel (*A*). Bars represent the mean number of positive cells per millimeter of retinal width. *Cnn2^−/−^* mice exhibited a reduced and sparser distribution of cone photoreceptor cells than WT controls. n (WT) = 8 and n (*Cnn2* KO) = 8 images. (*D*) Representative whole mount images of the cone arrestin marker in *Cnn2* KO mice. The labeling is concentrated at the OPL. Images were captured at 20× magnification. (Scale bar, 50 μm.) (*E*) Quantification of cone photoreceptor cell number in the OPL. The bar plot represents the mean number of cone arrestin-labeled cells counted per field of view (n=16 images pooled from 4 eyes per genotype). (*F*) Western blot for ARR3 expression in *Cnn2* KO mice. ARR3 is a cone photoreceptor-specific protein expressed in mouse retinas. (*G*) Quantitative analyses of relative protein expression levels of ARR3. n (WT) = 3 and n (*Cnn2* KO) = 3 samples. (*H*) Confocal image illustrating the expression levels of the rhodopsin marker. Images were captured at 40× magnification. (Scale bar, 20 μm.) (*I*) Quantification of rhodopsin intensity. The mean intensity ratio of rhodopsin^+^/DAPI^+^ labeling was calculated. n (WT) = 7 and n (*Cnn2* KO) = 7 images. (*J*) Representative images of the recoverin marker. Images were captured at 40× magnification. (Scale bar, 20 μm.) (*K*) Quantification of recoverin intensity. The mean intensity ratio of recoverin^+^/DAPI^+^ was calculated. n (WT) = 7 and n (*Cnn2* KO) = 6 images. (*L*) Western blot for Rhodopsin and Recoverin labeling in *Cnn2* KO and WT mice. The expression of Rhodopsin (rod photoreceptor cells) and Recoverin (photoreceptor cells) were detected. (*M* and *N*) Quantitative analyses of relative protein expression levels of (*M*) rhodopsin and (*N*) recoverin. n (WT) = 3 and n (*Cnn2* KO) = 3 samples. Bar plots throughout all panels are presented as the mean ± SEM. Two-tailed Student *t* tests were performed to compare each experimental group against the standard control, * *P* < 0.05, ** *P* < 0.01, *** *P* < 0.001. GCL, retinal ganglion cell layer; INL, inner nuclear layer; ONL, outer nuclear layer.

In addition to providing functional evidence for the role of *CNN2* in AMD pathogenesis, we investigated structural alterations in the retina. Outer retinal thickness was quantified using spectral domain optical coherence tomography (SD-OCT). Our results revealed a significant thinning of the photoreceptor layer in *Cnn2*^−/−^ mice at 3 and 5 mo of age (Fig. 4 *B* and *C* and *SI Appendix*, Fig. S17 *C* and *D*). Consistent with this structural decline, immunostaining for cone arrestin demonstrated diminished signals in 3-mo-old *Cnn2-*deficient mice (Fig. 5 *A*–*C*). Whole mount immunostaining corroborated the significantly decreased expression of cone arrestin at the outer plexiform layer (OPL) (Fig. 5 *D* and *E*). Rhodopsin and recoverin staining appeared relatively sparse compared to that of wild-type mice (Fig. 5 *H*–*K*).

To complement the immunostaining findings and quantitatively assess protein abundance, western blot analysis was conducted on whole retinal lysates from 3-mo-old mice (Fig. 5 *F*, *G*, and *L*–*N*). Quantitative densitometry confirmed a significant reduction in the protein levels of rhodopsin (*P* = 0.021) and cone arrestin (*P* = 0.015) in *Cnn2*-deficient mice compared to controls. Recoverin expression also exhibited a consistent downward trend, although it did not reach statistical significance (*P* = 0.095). The concordant structural and molecular alterations suggest that *Cnn2* depletion contributes to photoreceptor degeneration in the retina.

### Retinal Transcriptome Profiling in *Cnn2* Knockout Mice.

To characterize the molecular mechanisms underlying *Cnn2* deficiency, the transcriptional profile was systematically examined in the 3-mo-old *Cnn2*^−/−^ mice using full-thickness neural retina. A total of 436 differentially expressed genes (DEGs) were identified, with a false discovery rate (FDR) adjusted *P* < 0.05 and a fold change greater than 1.5, including 177 up-regulated genes and 259 down-regulated genes (*SI Appendix*, Fig. S18 *A* and *B* and Dataset S3). Consistent with the immunostaining and western blot findings, we observed a declined expression of photoreceptor-specific markers in 3-mo-old *Cnn2*^−/−^ mice. Specifically, RNA-seq data showed significant downregulation of cone markers *Arr3* (Cone Arrestin, fold change = 0.60, *P*_FDR_ = $2.7\times 10^{-5}$) and *Opn1mw* (M-opsin, fold change = 0.59, *P*_FDR_ = $4.6\times 10^{-5}$). Among the DEGs, *Egr1*, a critical transcriptional regulator involved in brain development, learning, and long-term neuronal plasticity (38), emerged as the most significantly downregulated gene. We found that the DEGs were significantly enriched for genes located within known AMD GWAS loci (8) (6.02-fold enrichment, *P* = 0.015), including *Abca7* (*P*_FDR_ = $2.8\times 10^{-6}$; AMD locus at chr19:1059243), *Rorb* (*P*_FDR_ = $3.8\times 10^{-9}$; AMD locus at chr9:76622068) and *Vegfa* (*P*_FDR_ = $1.6\times 10^{-4}$; AMD locus at chr6:43826627) (*SI Appendix*, Fig. S19). Gene set enrichment analysis (GSEA) of Kyoto Encyclopedia of Genes and Genomes (KEGG) pathways for 436 DEGs revealed suggestive enrichment in oxidative phosphorylation ($P=2.3\times 10^{-3}$*, P*_FDR_ =0.20) and neurodegeneration ($P=7.5\times 10^{-3}$ *, P*_FDR_ =0.26) (Dataset S3). This finding is consistent with transcriptomic and proteomic signatures previously reported in human AMD (39), suggesting that oxidative phosphorylation and neurodegenerative processes may contribute to the retinal phenotype associated with *Cnn2* deficiency.

By interrogating a published single-cell RNA-seq dataset from the mouse retina (40), we found that *Cnn2* was predominantly expressed in vascular-associated cell types, with the highest expression observed in endothelial cells and pericytes, whereas its expression was minimal in rod and cone photoreceptors (*SI Appendix*, Table S4 and Fig. S19). To further characterize cell-type associated alterations in *Cnn2* KO mice, we performed cell-type deconvolution to estimate the proportions of each retinal cell type using CIBERSORT (41). Dirichlet regression analysis revealed significant reductions in the inferred proportions of endothelial cells ($P=3.5\times 10^{-6}$), pericytes ($P=2.4\times 10^{-7}$), and rod photoreceptors ($P=7.4\times 10^{-4}$) in *Cnn2* KO mice compared to controls (*SI Appendix*, Table S5). These findings indicate that *Cnn2* deficiency is associated with alterations in both retinal vascular-associated cells and photoreceptors. Given the preferential expression of *Cnn2* in endothelial cells and pericytes (*SI Appendix*, Table S4 and Fig. S19), disruption of vascular or neurovascular homeostasis may represent one mechanism contributing to the photoreceptor degeneration and visual deficits observed in *Cnn2*^−/−^ mice. Nevertheless, the current data do not establish whether photoreceptor abnormalities arise directly from *Cnn2* deficiency within photoreceptors or indirectly through changes in vascular-associated or other retinal cell types. Cell-type-specific perturbation studies will therefore be required to delineate the temporal and causal contributions of these cell populations to disease pathogenesis.

Despite our findings in animal models, *CNN2* expression did not differ significantly between AMD and non-AMD control samples in human retina bulk RNA-sequencing datasets (42) (*SI Appendix*, Fig. S20). Nevertheless, *CNN2* expression was generally lower in AMD samples (P=0.15), with the downward trend appearing more pronounced in the macula region than the nonmacula region. This lack of statistical significance may reflect disease heterogeneity, the relatively small sample sizes, and the limited ability of bulk RNA sequencing to detect subtle or cell-type-specific changes. Larger AMD cohorts incorporating single-cell or spatial transcriptomic profiling will be needed to clarify the cellular and clinical contexts in which *CNN2* expression is altered.

## Discussion

In this study, we integrated AMD GWAS and eQTL data to prioritize candidate genes potentially involved in AMD susceptibility and subsequently evaluated their functional relevance in zebrafish and mouse models. Using the SMR and HEIDI framework with a blood eQTL dataset, we identified 9 putative AMD risk genes, 4 of which were further supported by an independent retinal eQTL dataset. In zebrafish larvae, knockdown of *cnn2* or *sarm1* resulted in ocular defects and impaired visual function. These phenotypes were substantially rescued by supplementation of the corresponding mRNAs, supporting the specificity of the observed effects. We subsequently focused on *CNN2* because it was supported by multiple layers of gene-prioritization evidence. *Cnn2*-deficient mice exhibited impaired photoreceptor function, decreased contrast sensitivity, thinning of the photoreceptor layers, and cone photoreceptor loss. Given the preferential expression of *Cnn2* in endothelial cells and pericytes, its deficiency may disrupt vascular or neurovascular homeostasis, potentially leading to secondary photoreceptor impairment and consequent retinal dysfunction. Collectively, these findings provide convergent genetic and experimental evidence supporting a role for *CNN2* in AMD-related retinal dysfunction, from in silico gene prioritization to in vivo functional characterization.

*CNN2* encodes calponin 2, a member of the calponin family of actin-binding proteins (43). Calponin 2 is expressed in smooth muscle and nonmuscle cells, including fibroblasts and endothelial cells, where it regulates a range of actin cytoskeleton-dependent processes. It has been shown to modulate cell proliferation in pathological contexts such as prostate cancer (44) and acute kidney injury (45). Calponin 2 also regulates the motility of primary fibroblasts and macrophages, as well as angiogenic cell migration and platelet adhesion (46). In addition, *CNN2* has been implicated in immune function, with cell-specific or systemic knockout producing anti-inflammatory effects in models of inflammatory arthritis (47), arterial atherosclerosis (48), and calcific aortic valve disease (49). More recently, calponin 2 has been reported to contribute to phagophore formation through the regulation of actin filaments, a process important for the clearance of damaged lysosomes and the maintenance of cellular homeostasis, which has implications in neurodegenerative processes (50, 51).

The study has some limitations. First, our discovery phase relied on a large blood eQTL dataset (n=2,765), while the replication phase utilized a relatively small retinal eQTL dataset (n=406), following the approach suggested by a previous study (52). This design balances statistical power against tissue specificity but may miss genes with retina-specific *cis*-eQTL effects that are absent in blood. Furthermore, the limited sample size of current retinal eQTL resources inherently constrains the statistical power of SMR tests, potentially leading to false negatives for risk genes with modest effect sizes. Differences between LD reference panels may also introduce technical noise into the HEIDI tests. Previous AMD transcriptome-wide association studies using liver eQTLs (53) and eQTLs from 27 tissues (54) have identified numerous associated genes, considering the systemic nature of AMD pathology. Thus, beyond gene discovery, a major challenge is to establish efficient functional screening strategies that can determine which prioritized genes are biologically relevant to AMD.

Second, the regulatory mechanisms linking AMD-associated variants to their effector genes remain incompletely understood. Based on human RPE/choroid open-chromatin data, we hypothesize that fine-mapped variants at the *CNN2* locus may alter promoter activity and thereby influence *CNN2* transcription (Fig. 1*B* and *SI Appendix*, section 3 and Fig. S12 and Dataset S2). Further mechanistic studies integrating single-cell chromatin accessibility, transcriptomic, and three-dimensional genome data from the human retina, together with single-cell perturbation experiments, will be required to test this hypothesis. Although fine-mapping causal variants remains important, identifying the corresponding effector genes is essential because multiple variants may converge on the same gene or pathway. Biological evidence related to retinal vasculature, inflammation, lipid metabolism, and shared mechanisms with other age-related neurodegenerative diseases may therefore help prioritize candidates for functional validation. Notably, *CNN2*, *PILRB*, and *ABCA7* are located at risk loci associated with late-onset Alzheimer’s disease (AD), suggesting potential genetic or biological overlap between AMD and AD. Nevertheless, given the complexity of gene regulation and the limitations of current functional assays, contributions from other prioritized genes cannot be excluded.

Third, zebrafish and mouse models have inherent limitations for studying AMD. Although they provide valuable functional evidence, findings from these models should be regarded as supportive rather than definitive, as they cannot fully recapitulate the extensive genetic variation, epistatic effects, and gene–epigenome interactions underlying AMD in humans. Because MO-mediated knockdown is most effective during the first few days of zebrafish development and declines rapidly with age (55), larval zebrafish were used, precluding assessment of age-dependent phenotypes. Moreover, neither zebrafish nor mice possess a macula or foveal centralis, the principal sites affected in human AMD, and prominent drusen were not observed within the time frame of this study. Although our study identified robust retinal phenotypes in *Cnn2*^−/−^ mice up to 5 mo of age, longitudinal analyses extending to 12 to 18 mo will be important for determining the long-term and age-dependent consequences of *Cnn2* deficiency in the retina. However, common AMD-associated regulatory variants typically exert modest effects on gene expression (14), whereas MO-mediated knockdown or complete gene knockout represents a substantially stronger perturbation and may therefore reveal disease-relevant consequences at earlier developmental or adult stages. Primate models would provide greater anatomical relevance because they possess a macula, but their use is limited by substantial time and cost requirements. Zebrafish and mice therefore remain practical models for efficient screening and functional validation, as reflected by their extensive use in AMD research (11, 56).

In summary, by integrating AMD GWAS and eQTL data with cross-species functional characterization, our study identifies *CNN2* as a biologically relevant AMD susceptibility gene. This framework bridges human genetic associations with retinal phenotypes and provides a broadly applicable strategy for prioritizing candidate genes and investigating disease-relevant mechanisms in complex human disorders.

## Materials and Methods

### Data Used for the Integrative Analysis.

The GWAS summary data used in this study were obtained from an AMD GWAS meta-analysis (6) (http://amdgenetics.org/), consisting of 16,144 individuals with advanced AMD and 17,832 controls, predominantly of European ancestry. The dataset included approximately 12 million SNPs, with SNP effects reported as log odds ratios. Because allele-frequency information was unavailable in the original dataset, we estimated allele frequencies using data from the UK Biobank of European ancestry (16).

The eQTL summary statistics were obtained from the CAGE dataset (14), consisting of 36,778 transcriptomic traits and approximately 8 million SNPs from 2,765 peripheral blood samples, primarily of European ancestry. Transcript abundance was quantified using Illumina gene expression arrays. To validate the findings in retinal tissue, *cis*-eQTL summary data were obtained from the Eye Genotype Expression (EyeGex) database (https://gtexportal.org/home/datasets), which included 406 postmortem retinas with approximately 9 million SNPs and 15,124 gene expression traits (17). For cross-tissue replication, we used the GTEx v7 dataset, which provided *cis*-eQTL summary statistics from 48 human tissues (https://yanglab.westlake.edu.cn/software/smr/#DataResource; https://yanglab.westlake.edu.cn/software/smr/). Transcript abundance in both EyeGex and GTEx was measured using RNA-sequencing. Across all three eQTL datasets, effect sizes were reported in SD units of transcript abundance.

### SMR and HEIDI Tests for Pleiotropic Association.

SMR and HEIDI analyses were developed to identify genes whose expression levels are associated with a complex trait because of pleiotropy or causality (i.e., the trait and gene expression are associated due to the same set of causal variants at a locus) (15). The SMR test takes the top associated *cis*-eQTL of a gene as an instrumental variable to examine the association between a transcript (as an exposure) and AMD (as an outcome). The SMR estimate represents the ratio of the estimated effect of the instrumental variant on the disease outcome (GWAS effect) to its effect on transcript abundance (eQTL effect). The SE of the SMR estimate is calculated using the delta method, and statistical significance is assessed using a Wald test. To distinguish SMR associations resulting from linkage (i.e., the trait and gene expression are associated due to a distinct set of causal variants in LD), the HEIDI analysis incorporates multiple SNPs within a *cis*-eQTL region to test against the null hypothesis that the trait is associated with gene expression because of the same set of underlying causal variants (pleiotropy/causality). Under this null hypothesis, the SMR effects estimated using different eQTL SNPs in the *cis* region are expected to be consistent. Significant heterogeneity in SMR effects across SNPs in LD with the top *cis*-eQTL, defined as $P_{\mathrm{HEIDI}}<0.05$, suggests linkage and leads to the exclusion of these associations from further analysis. Precomputed LD correlations for the HEIDI test were derived from genotype data of 20,000 unrelated participants of European ancestry randomly selected from the UK Biobank. Following the default settings, the *r^2^* thresholds were set to an upper limit of 0.9 and a lower limit of 0.05.

### Morpholino, mRNA Rescue, and Overexpression Experiments.

For the *cnn2* mRNA rescue experiment, zebrafish cDNA was generated from total RNA using RT-PCR with a full-length *cnn2* fragment as a template. For the *sarm1* mRNA rescue and *bloc1s1* overexpression experiments, we obtained specific cDNA from the pUC57 vector cloned into template cDNA sequences of *sarm1* or *bloc1s1*, which were obtained through oligoribonucleotide synthesis (Sangon Biotech, Shanghai, China). The amplification primers of *cnn2* included the forward (5′-TAATACGACTCACTATAGGGGCCACCATGTCTTCGCAG-3′) and the reverse (5′-TTAGTAATCTTGGCCGTCGTCCTGATAGC-3′); *sarm1* included the forward (5′-TAATACGACTCACTATAGGGGCCACCATGTTTTTGTCCCTCG-3′) and the reverse (5′-CTACTTCTTTTGTGGCTCTTTTTTGTCCG-3′); *bloc1s1* included the forward (5′-TAATACGACTCACTATAGGGGCCACCATGCTCTCGCGG-3′) and the reverse (5′-TCATGTGGATGCCGGCTGGAC-3′); they all flank the T7 promoter sequence (5′-TAATACGACTCACTATAGGG-3′) and the Kozak sequence. The PCR template DNA was purified using the QIAquick PCR Purification Kit (Qiagen, Germany). Capped and tailed full-length mRNA was synthesized using an mMESSAGE mMACHINE™ T7 ULTRA Transcription Kit (Invitrogen, Carlsbad, CA), followed by purification using the RNeasy Mini Kit (Qiagen, Germany) according to the manufacturer’s protocols. The rescue mRNAs were coinjected with MO into one-cell-stage embryos. For overexpression, the corresponding mRNAs were injected into one- to two-cell-stage embryos.

### Measurement of Ocular Parameters and Body Length.

The ocular parameters and body length in embryos at 3 d postfertilization (dpf) were assessed using a stereomicroscope (SZX16, OLYMPUS, Japan). Images capturing the vertical and lateral views of each larva were recorded using a microscopic camera. The built-in software (Olympus cellSens standard, v1.14) was employed for quantifying axial length, eye area, and body length. Each experimental group comprised 10 to 15 larvae, and the experiments were replicated three times. Statistical analysis was performed using Student’s *t* test to compare the control and treatment conditions for each phenotype. The obtained data were processed and visualized using R software (v3.5.3) and the “ggplot2” package (v3.1.0).

### Visual Behavior Experiments.

The visual motor response (VMR) of 5-dpf larvae was measured using a Zebrabox (VMR machine ViewPoint 2.0, France) to evaluate both light-on and light-off responses. Each treatment group consisted of 12 larvae, which were individually placed in separate wells of a 96-well plate with adequate water to allow unrestricted movement. The Zebrabox protocol involved the following steps: 1) a 3-h period of dark adaptation, 2) 30 min of ON light exposure, 3) 30 min of OFF light, and 4) repetition of steps 2 and 3 for a total of three cycles. Motor activities were recorded every second, and a 150-s duration (30 s before and 120 s after light switching) was analyzed to evaluate the visual motor activities of the larvae. The aggregate data from three repetitions were compiled and visualized in the figures. The area under the curve, calculated using the R package MESS (v1.0), was used to quantify the overall motor response during the 150-s light-transition window. For each group, 12 randomly selected larvae were subjected to the experiments, which were routinely performed between 11:00 and 17:00.

### Real-Time Quantitative PCR.

Zebrafish eyes were isolated from both the control and experimental groups (n=30 pairs for each group) at 3 dpf. Total RNA was extracted using TRIzol reagent (Invitrogen Life Technologies, Carlsbad, CA). The concentration of RNA was determined using a NanoDrop instrument (NanoDrop Technologies, Thermo). According to the manufacturer’s instructions, 500 ng of purified RNA was used to generate cDNA using PrimeScript reverse transcriptase (TaKaRa, Dalian, China). Real-time quantitative PCR was performed using the FastStart Universal SYBR Green Master (Roche Applied Science, Mannheim, Germany). Specific primers used in the PCR can be found in *SI Appendix*, Table S3. The relative expression levels were measured using the StepOnePlus™ Real-time PCR System (Life Technologies, Carlsbad, CA), and the 2^−ΔΔCT^ method was applied for fold change calculation, with the standard MO control serving as the reference. All experiments were performed in triplicate.

### Immunohistochemistry.

For immunostaining, zebrafish morphants and control larvae at 3 dpf were fixed in 4% paraformaldehyde and rinsed with 15% and 30% sucrose in phosphate-buffered saline (PBS) for dehydration. The zebrafish samples were then directly frozen in Richard-Allan Scientific™ Neg-50™ embedding medium. Cryosections of 18-μm thickness were obtained from the zebrafish eyes and stained with zpr-1, zpr-2, and zpr-3 zebrafish-specific antibodies (1:400; provided by ZFIN) overnight at 4 °C. Incubation with Alexa Fluor 594 anti-mouse secondary antibodies (1:400; provided by ZFIN) in blocking solution was carried out for 2 h at room temperature. Nuclei were counterstained with DAPI (4,6-diamidino-2-phenylindole). Following mounting of coverslips, gene expression and retinal architecture were examined using a confocal microscope (TCS SP8, Leica, Germany).

### Gene Prioritization.

In the gene-based association test, we employed MAGMA (v1.10) (30) and GCTA-mBAT-combo (v1.94.1) (31), setting Bonferroni-corrected *P* value thresholds at 0.05/18071 and 0.05/18742, respectively. We conducted FUSION-TWAS (32) analyses using GTEx v8 eQTL data (57). We utilized the “CARMA” R package (v1.0) (33) to perform statistical fine-mapping. For each flanking 1 Mb region harboring a significant locus, LD matrix was calculated from 50 K unrelated UKB-EUR individuals. Publicly available human RPE/choroid single-cell ATAC-seq data (GEO accession ID: GSE262151) (34) were used to assess whether fine-mapped variants with posterior inclusion probability (PIP) > 0.1 overlapped open chromatin regions. Pseudobulk accessibility profiles were generated separately for each annotated cell type by pooling ATAC-seq fragments from all cells assigned to that cell type. Fragment midpoints were counted in nonoverlapping 500-bp genomic bins surrounding each variant, and the accessibility signal in the variant-containing bin was examined across cell types. We additionally applied the Activity-by-Contact (58) (ABC) model as a regulatory gene-prioritization approach. The ABC model predicts enhancer–gene relationships by integrating enhancer activity with chromatin contact frequency between candidate enhancers and target-gene promoters. We used a published ABC prediction map comprising 6,316,021 enhancer-gene links involving 269,539 unique enhancers and 23,219 expressed genes across 131 cell and tissue types. Fine-mapped variants were intersected with ABC-annotated enhancers, and genes linked to the overlapping enhancers with an ABC score > 0.02 were prioritized as putative regulatory targets.

### Generation of *Cnn2* Knockout Mice.

Animal care adhered strictly to the guidance outlined in the Statement for the Use of Animals in Ophthalmic and Vision Research, as established by the Association for Research in Vision and Ophthalmology (ARVO). All animal experiments were conducted with the approval of the Animal Care and Use Committees of Wenzhou Medical University. The *Cnn2* knockout mice were obtained from Cyagen (Cyagen, Suzhou, China). In detail, the sgRNAs were designed to target the first and seventh exons. Coinjection of these sgRNAs and Cas9 mRNA was performed in zygotes of C57BL/6 J mice to obtain the chimeras. Subsequently, homozygotes were obtained through a series of backcrossing, hybridization, and PCR analysis steps. Both sexes were included in all experiments.

### Sanger Sequencing of *Cnn2* Knockout Mice.

The *Cnn2* gene sequence was amplified using PCR and subsequently verified by Sanger sequencing. The primers utilized in the amplification process were as follows: forward primer: 5′-GATACCCTGCTAAGCCTGTGTGC-3′, reverse primer 1: 5′-GAGCTGGAG CACTGATGGATGAG-3′, and reverse primer 2: 5′-ATGTCCCCTGTCTGGTGGGTCAAT-3′. The DNA band observed for homozygotes was 560 bp, while the wild type exhibited a band of 727 bp. Heterozygotes displayed both bands concurrently. The NCBI Reference Sequence: NM_007725.2; Ensembl: ENSMUSG00000004665.

### Real-Time Quantitative PCR for *Cnn2* Knockout Mice.

Retinas were dissected from 3-mo-old mice, and total RNA was isolated using TRIzol reagent (Life Technologies). RNA was quantified using the NanoDrop 2000 Spectrophotometer (Thermo Fisher Scientific, Waltham, MA). Then, 1 μg of total RNA was reverse transcribed into cDNA with M-MLV reverse transcriptase (Cat. M1705, Promega) and random primers (Cat. C1181, Promega). Then, 10 ng of cDNA was mixed with SYBR Green PCR Master Mix (Roche, Basel, Switzerland) and 1 μM target gene primers in the 20-μL mixture solution. The standard protocol of qRT-PCR was performed by the LightCycler 96 Real-Time PCR System (Roche, Basel, Switzerland). The primers used for *Cnn2* mRNA identification were as follows: forward primer: 5′-AACAAGGGCCCGTCCTAC-3′, reverse primer: 5′-TTCCTTTTGGGGGTCATATTT-3′.

### ERG.

ERGs were recorded using the Ganzfeld-field ERG system (Pleasanton, CA). The experiment was performed in a fully darkened room, with the mice subjected to a dark adaptation period of over 3 h. Prior to the ERG measurements, mouse pupils were dilated with 0.5% tropicamide for 5 min, and anesthesia was induced using 1.12% pentobarbital sodium. To maintain corneal moisture, ofloxacin eye ointment (Dicolol) was applied. The reference electrode was carefully positioned on the skin between the two eyes, while the ground electrode was inserted into the tail. The stability of ERG waveforms was verified by checking the chart mode. ERG stimulations were provided by an LED light source. The ERG responses of the mice were measured at P1m, P3m, and P5m. The scotopic ERGs were recorded at 0.01, 3.0, and 10.0 cd^.^s/m^2^. Photopic ERGs were recorded after steady background illumination for 5 min. Photopic ERGs were recorded at 3.0 cd^.^s/m^2^.

### Fundus Photographs (FP) and High-Resolution SD-OCT.

FP and SD-OCT images of *Cnn2* knockout mice were obtained using the Micron-IV retinal imaging system (Pleasanton, CA). Prior to imaging, the mice underwent pupil dilation with 0.5% tropicamide for 5 min, followed by anesthetization with 1.12% pentobarbital sodium. Ofloxacin eye ointment (Dicolol) was applied to maintain corneal moisture. The mice were positioned facing the camera for image capture. Images were taken along the vertical retina, crossing the optic nerve, with a total of 50 images acquired to generate the final averaged SD-OCT image. Imaging procedures were conducted at P1m, P3m, and P5m. The thickness of retinal layers was calculated by the Micron-IV retinal imaging system.

### OKR.

Mice at P3m were positioned on a centrally placed platform within a square apparatus, while vertical black and white stripe patterns rotated around the animal at a speed of 12°/s. Mouse behaviors were captured using a top-mounted television camera within the testing chamber. OptoDrum software (Striatech, Germany) was employed for the analysis of optomotor responses in mice. The software enabled the configuration of a gradient of test parameters, automatically evaluating the OKR of mice and determining subsequent test parameters. A positive response was defined as a head movement in the direction of the moving stripes. We assessed the maximum number of stripes that elicited a reflexive response from the mice at 100% contrast, as well as the lowest contrast at which responses were observed for 60 and 120 stripes, respectively.

### Retinal Immunostaining.

The mice were thoroughly perfused with Dulbecco’s phosphate-buffered saline (DPBS) prior to tissue dissection. The eyecup was extracted from mice and fixed in 4% paraformaldehyde for 1 h. Subsequently, the eyecup underwent dehydration using 15% and 30% sucrose solutions, followed by embedding in optimum cutting temperature compound and freezing at −80 °C. The frozen sample was then sectioned at a thickness of 12 μm and placed onto slides. To prepare the slides for immunostaining, a rinsing step with 0.01 M PBS was performed three times, with each rinse lasting for 10 min. Following rinsing, the slides were blocked in a PBS solution containing 4% Bovine Serum Albumin and 0.5% Triton X-100 for 60 min. The primary and secondary antibodies were diluted in a PBS solution containing 1% Bovine Serum Albumin and 0.5% Triton X-100. The slides were then incubated with the primary antibodies overnight at 4 °C. After incubation, the slides were washed three times with 0.01 M PBS and subsequently incubated with the secondary antibodies for 60 min at room temperature.

The primary antibodies and their respective dilutions used in this study were as follows: mouse anti-Cnn2 (1:200, Merck, C2687), rabbit anti-CD31 (1:200, Abcam, ab222783), Isolectin B4 (1:50, Vector Labs, B-1205), rabbit anti-Recoverin (1:200, Millipore, AB5585), mouse anti-Rhodopsin (1:1000, Invitrogen, MA1-722), rabbit anti-Cone Arrestin (1:200, Millipore, AB15282), rabbit anti-M-opsin (1:200, Millipore, AB5405), rabbit anti-S-opsin (1:200, Millipore, AB5407), rabbit anti-Iba1 (1:200, Abcam, ab178846), rabbit anti-Glutamine Synthetase (GS) (1:200, Abcam, ab73593), mouse anti-CD68 (1:200, Santa Cruz, sc70761), and mouse anti-Synaptophysin (1:200, Santa Cruz, sc-12737).

The secondary antibodies and their respective dilutions used in this study were as follows: Fluorescein Avidin D (1:50, Vector Labs, A-2001-5), donkey anti-mouse IgG conjugated to Alexa Fluor 594 (1:200, Invitrogen, A21203), donkey anti-rabbit IgG conjugated to Alexa Fluor 488 (1:200, Invitrogen, A21206), and donkey anti-rabbit IgG conjugated to Alexa Fluor 594 (1:200, Invitrogen, A21207). The 4,6-diamidino-2-phenylindole (DAPI, 1:3000, Invitrogen, D1306) stained the cell nuclei. Images of retinal immunostaining were acquired using an Olympus SpinSR10 laser scanning confocal microscope (Olympus, Tokyo, Japan).

### Whole Mount Immunostaining.

For retinal whole-mount immunostaining, the eyecups were extracted from mice and fixed in 4% paraformaldehyde for 1 h. Subsequently, the retinas were incubated in a solution containing 4% Bovine Serum Albumin and 0.5% Triton X-100 at 4 °C for 1 d. This was followed by incubation in primary antibody solution (1% Bovine Serum Albumin and 0.5% Triton X-100) for 3 d, with subsequent washing using PBS prior to the secondary antibody incubation (1% Bovine Serum Albumin and 0.5% Triton X-100) for 1 d. Whole mount immunostaining images were captured using an Olympus SpinSR10 laser scanning confocal microscope (Olympus, Tokyo, Japan).

The primary antibodies and dilutions were as follows: rabbit anti-Cone Arrestin (1:200, Millipore, AB15282), rabbit anti-M-opsin (1:200, Millipore, AB5405), and rabbit anti-S-opsin (1:200, Millipore, AB5407). The secondary antibodies and dilutions were as follows: donkey anti-rabbit IgG conjugated to Alexa Fluor 488 (1:400, Invitrogen, A21206), and donkey anti-rabbit IgG conjugated to Alexa Fluor 594 (1:400, Invitrogen, A21207).

### Quantitative Analysis of Immunostaining Images.

Quantitative image analysis was performed using ImageJ (v1.54f, NIH, Bethesda, MD) for cell counting and intensity measurements, and Olympus OlyVIA (v3.2.1, Olympus, Tokyo, Japan) for specific measurements of tissue dimensions. Consistent imaging parameters (laser power, gain, and exposure) were maintained throughout image acquisition and were not adjusted during subsequent analysis to ensure data comparability.

#### Cone photoreceptor cell count.

Cone photoreceptor cells labeled in frozen sections were counted manually and the retinal tissue width was measured in the Olympus OlyVIA software. The number of labeled cones per millimeter of tissue width was calculated as cone photoreceptor cell count/retinal tissue width (mm). Whole mount images were quantified using the Analyze Particles feature in ImageJ. Images of equal size were converted to 8-bit grayscale and processed by adjusting the threshold to accurately isolate the labeled cone photoreceptor cells. The Analyze Particles model was then used to count the number of positive markers, with a uniform size exclusion parameter set to 0.0001–infinity. The total number of cone photoreceptor cells per image was recorded.

#### Intensity ratio.

Fluorescence intensity ratios were calculated to normalize signal intensity variations across different tissue samples. The average fluorescence intensity of the positive staining (e.g., Rhodopsin or Recoverin) and the nuclear counterstain (DAPI) were measured within the photoreceptor layer using ImageJ. The relative intensity ratio was calculated to control for slice-to-slice imaging variability: Relative intensity ratio = Rhodopsin^+^ or Recoverin^+^ intensity/DAPI^+^ intensity.

### Western Blot.

The mouse eyeball was obtained and the cornea and lens were removed in DPBS. The retinal tissue was isolated via blunt dissection from the underlying choroid. Retinal tissues were homogenized in a lysis buffer supplemented with 1× Protease Inhibitor Cocktail (Roche, Cat. 04693132001). Total protein concentrations were determined using a Bicinchoninic Acid (BCA) Protein Assay Kit (Invitrogen, Cat. 23225) according to the manufacturer’s instructions. Equal amounts of protein were separated by SDS-PAGE using 10% polyacrylamide gels (Beyotime, Cat. P0012A) and subsequently transferred onto nitrocellulose membranes. The nitrocellulose membranes with proteins were blocked in 5% skim milk for 1 h at room temperature. The membranes were incubated with primary antibodies overnight at 4 °C. The membranes were washed three times with 0.5% Tween-TBS for 10 min each, incubated with secondary antibodies for 1 h at room temperature, and washed three times with 0.5% Tween-TBS for 10 min each. The primary antibodies were diluted in antibody diluent (Beyotime, Cat. P0023A), and secondary antibodies were diluted in 5% skim milk. The chemiluminescence substrate (Millipore, Cat. WBKLS0500) was applied on the nitrocellulose membranes for 2 to 3 min. A chemiluminescence imaging system (Tanon, Shanghai, China) was used to image the protein bands. ImageJ software (v1.54f, NIH, Bethesda, MD) was used for relative quantification of western blot results. The integrated density of each protein band was measured in this study. The integrated density ratio of target protein/internal reference protein was calculated.

The primary antibodies and their respective dilutions used in this study were as follows: rabbit anti-Arr3 (1:500, Invitrogen, PA5-75301), rabbit anti-Recoverin (1:500, Invitrogen, PA5-110287), rabbit anti-Rhodopsin (1:500, Bioss, bs-19872R), and rabbit anti-GAPDH (1:2,000, Cell Signal Technology, 97166S). The secondary antibodies and their respective dilutions used in this study were as follows: goat anti-rabbit HRP (1:2,000, Cell Signal Technology, 7074S).

### Transcriptional Profiling and Bioinformatic Analyses.

Retinas were collected, and total RNA was extracted using TRIzol Reagent (Life Technologies). The concentration of the extracted total RNA was quantified using the NanoDrop 2000 (Thermo Fisher Scientific, Waltham, MA). The cDNA library was constructed, and high-throughput sequencing was performed using the Illumina NovaSeq 6000 sequencing platform (Illumina, San Diego, CA). To align the obtained sequencing reads, the reference genome GRCm39 was utilized with the HISAT2 alignment tool (59). The feature counts, indicating the number of reads mapped to each gene, were determined using featureCounts (60). Differential gene expression analysis between the knockout (KO) mice and the control group was conducted using the DESeq2 package (v1.40.2). Genes were considered differentially expressed if they exhibited a fold change >1.5 and a false discovery rate (FDR) < 0.05. A one-sided Fisher’s exact test was used to determine whether differentially expressed genes were enriched among genes located at known AMD GWAS loci (8). Gene-set enrichment analysis was performed using the Kyoto Encyclopedia of Genes and Genomes (KEGG) pathways. This analysis was carried out using the clusterProfiler (v4.8.2). To estimate the relative proportions of retinal cell types from bulk RNA-seq samples, we employed the CIBERSORT (v0.1.0) analytical framework (41). A reference signature matrix was constructed using a publicly available mouse retinal single-cell RNA-sequencing (scRNA-seq) dataset (40). Deconvolution was performed using 1,000 permutations to ensure statistical robustness. The resulting cell-type fractions were compared between *Cnn2* knockout and wild-type groups using a Dirichlet regression model to account for the compositional nature of deconvolution data, implemented in the DirichletReg R package (v4.3.3). We applied the Bonferroni correction to all *P* values derived from the Dirichlet regression model. Statistical significance was defined as a *P* value less than $4.2\times 10^{-3}$ (i.e., 0.05/12). *CNN2* expression in human retinal samples was quantified as normalized reads per kilobase per million mapped reads using the publicly available dataset (GEO accession ID: GSE135092) (42). Expression was compared between AMD and non-AMD control samples across all samples and after stratification by tissue type (neural retina or RPE/choroid), retinal region (macular or nonmacular), and the combination of tissue type and retinal region. Differences between AMD and non-AMD control samples were assessed using two-sided Mann–Whitney *U* tests.

## Supplementary Material

Appendix 01 (PDF)

Dataset S01 (XLSX)

Dataset S02 (XLSX)

Dataset S03 (XLSX)

## Data Availability

Study data are included in the article and/or supporting information.
