# Supplementary material for: Genetic evidence and cross-species functional characterization implicate CNN2 in age-related macular degeneration susceptibility
Source: Proc Natl Acad Sci U S A. 2026 Sep 2;123(36):e2533682123. doi: 10.1073/pnas.2533682123 (PMC13552911; doi:10.1073/pnas.2533682123)
Supplement: Supplementary file 1 [file pnas.2533682123.sapp.pdf]

PNAS

Supporting Information for

Genetic evidence and cross-species functional characterization implicate *CNN2* in age-related macular degeneration susceptibility

Fei-Fei Cheng[1#], Hao Mou[2#], Zhenyu Liu[2], Chang-Jun Zhang[2], You-Yuan Zhuang[3], Zhen Wu[3], Xin-Ran Wen[3], Angli Xue[4,5,6], Xiao Zhang[2], Jian Yang[1,4,7,*], Zi-Bing Jin[2,8,*]

Zi-Bing Jin and Jian Yang
Email: jinzb502@ccmu.edu.cn, jian.yang@westlake.edu.cn

This PDF file includes:

- Supporting text
- Figures S1 to S20
- Tables S1 to S5
- Legends for Datasets S1 to S3
- SI References

Other supporting materials for this manuscript include the following:

- Datasets S1 to S3

Supporting Information Text

Section 1: SMR analysis using the latest GWAS and eQTL datasets.
At the time of the initial investigation (Sept 2019; as documented in our bioRxiv preprint (1)), this dataset represented the largest sample size for AMD GWAS and eQTL data. In light of subsequent advancements in the field, which have yielded significantly larger sample sizes for AMD GWAS (2) and blood-derived eQTL datasets (3), we sought to determine the continued robustness of our original findings.

To this end, we performed a replication analysis utilizing the most current large-scale datasets. Since the complete GWAS summary statistics for the largest published GWAS analysis are not publicly accessible, we conducted a GWAS meta-analysis to achieve a comparable statistical power. The meta-analysis synthesized data from the IAMDGC AMD-2016 GWAS ($n_{eff} = 33{,}892$) (4), the UK Biobank AMD GWAS ($n_{eff} = 108{,}042$) (5, 6), GERA AMD GWAS ($n_{eff} = 53{,}888$) (6, 7), FinnGen AMD GWAS ($n_{eff} = 294{,}394$) (8), MVP AMD GWAS ($n_{eff} = 45{,}523$) (9), reaching a total effective sample size of 535,739. For the eQTL component, we used eQTLGen consortium data (3), a large-scale meta-analysis comprising approximately 31,684 individuals to characterize the genetic architecture of gene expression in human blood. The replication demonstrated a high degree of consistency with the primary analysis using the same SNP instruments (Dataset S1). Notably, the candidate gene *CNN2* maintained its significant association with $P_{smr} = 5.99 \times 10^{-7}$.

Section 2: The phenotypes of the *Sarm1*-knockout mice.
Our preliminary experiments examined the association between *SARM1* and AMD-relevant phenotypes using *Sarm1*-knockout mice. Animal care procedures adhered to the guidelines outlined in the Statement for the Use of Animals in Ophthalmic and Vision Research, as established by the Association for Research in Vision and Ophthalmology (ARVO). The Animal Care and Use Committees of Wenzhou Medical University provided approval for all animal experiments conducted. *Sarm1* knockout mice were obtained from the Jackson laboratory (Jackson, ME, USA, Stock No. 018069). In this mouse strain, the knockout targeted the regions spanning from exon 3 to exon 6 (*SI Appendix*, Fig. S8 *A*). Both male and female mice were utilized equally in the experiments. The *Sarm1* gene sequence was amplified through PCR, and Sanger sequencing was employed to verify the sequence accuracy (*SI Appendix*, Fig. S8 *B*). The following primers were utilized: forward primer: 5'-GAAATGCATGGAGGGGTTG -3', reverse primer 1: 5'- TGTGGTTTCCAAATGTGTCAG -3', and reverse primer 2: 5'- CCACCAAACGTGTCCAATC -3'. The homozygous genotype exhibited a DNA band of was 248 bp, the wildtype genotype displayed a band of 302 bp, while the heterozygous genotype exhibited both bands. The NCBI Reference Sequence: NM_001168521.1; Ensembl: ENSMUSG00000050132.

The ERG response of *Sarm1*$^{-/-}$ mice did not exhibit a significant difference compared to the control group, with the exception of compromised b-wave amplitudes observed during the photopic response at stimulus intensities of 3.0 and 4.0 log(cd·s/m^2) (*SI Appendix*, Fig. S9). Immunostaining experiments revealed relatively sparse cone markers (M-opsin and S-opsin) in the *Sarm1* KO mice, especially in the middle region (*SI Appendix*, Fig. S10). In addition, comprehensive evaluations using color fundus photography, fundus fluorescent angiography, and OCT did not reveal any apparent structural alterations in the *Sarm1*$^{-/-}$ mice (*SI Appendix*, Fig. S11).

Section 3: Additional genetic evidence linking AMD with *CNN2*

To explore additional genetic evidence linking AMD with *CNN2*, we employed multiple layers of genetic and genomic evidence. To aggregate SNP-level associations into biologically interpretable units, we first conducted gene-based association tests using MAGMA (v1.10) (10) and mBAT-combo (11) implemented in GCTA (v1.94.1) (12). These complementary approaches allow for the joint analysis of all genetic variants within and surrounding the *CNN2* locus. *CNN2* exhibited robust and highly significant association signals across the both gene-centric methods, yielding P-values of 2.2×10^{-7} for MAGMA and 3.7×10^{-9} for mBAT-combo. This consistency across independent statistical models underscores that the *CNN2* locus is significantly enriched with AMD risk-associated variation, prioritizing it as a high-confidence susceptibility gene.

We then explore the fine-mapped genetic variants corresponding regulatory support. Utilizing the CARMA method (13), we identified likely causal variants (posterior inclusion probability, PIP>0.1) within each GWAS locus. In the GRCh38 assembly, rs10422209 (CARMA PIP=0.40), approximately 45 bp downstream of the *CNN2* transcription start site (TSS; chr19:1,026,274). Thus, rs10422209 lies within the immediate promoter-proximal/5′ -UTR region of *CNN2* (Fig. 1 *B*), annotated by ENCODE as a proximal enhancer-like signature. Established regulatory maps — including activity-by-contact (ABC) model (14), EpiMap (15) and RoadMap (16) — demonstrate strong regulatory interactions between these fine-mapped variants and *CNN2*. The ABC score is 0.25 in the H1-drived neuronal progenitor cultured cells for the genetic variant of rs10422209, significantly exceeding the established high-confidence threshold of 0.02. This indicates that this regulatory element contributes approximately 25% of the total predicted regulatory input for *CNN2*, prioritizing it as a key *cis*-regulatory driver of *CNN2* expression. The ABC score linking rs10422209 and *CNN2* ranged from 0.10 to 0.25 across other cell types, suggesting its extensive and robust regulatory effects. To assess the regulatory potential of *CNN2*-associated variants in AMD-relevant retinal tissues, we examined cell-type-resolved scATAC-seq data from human RPE/choroid. rs10422209 falls within a locally accessible chromatin region in the RPE pseudo-bulk profile, with 193 fragment midpoints detected in the 500-bp bin containing the variant. Accessibility at this locus was also observed in fibroblasts and endothelial cells (*SI Appendix*, Fig.S12). These findings support a promoter-proximal regulatory context for rs10422209 in human RPE/choroid.

To bridge the gap between genetic associations and functional genes, we performed a transcriptome-wide association study (TWAS) integrating AMD GWAS summary statistics with GTEx eQTL weights from the whole blood. *CNN2* showed a strong negative association $P = 1.9 \times 10^{-8}$, indicating that genetically predicted lower expression of *CNN2* in whole blood is associated with increased risk of AMD. Such convergent signals across multiple multi-omics integration methods (e.g., SMR and TWAS) support the role of *CNN2* as a putative causal candidate at this locus.

Figures

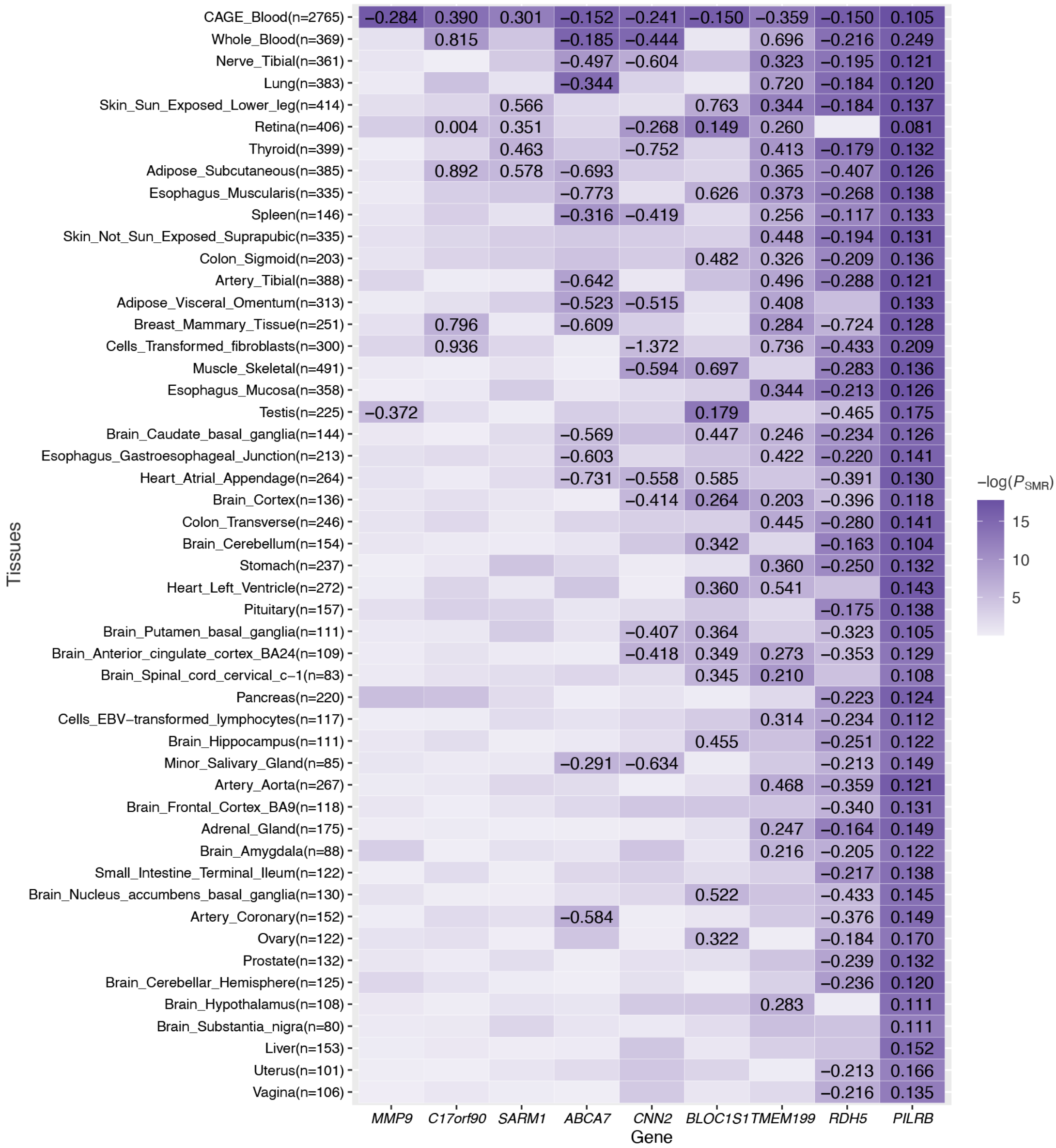

Fig. S1. Heatmap of SMR results of the nine prioritized genes across multiple tissues. Each row represents a prioritized gene, and each column represents one tissue. The intensity of the color scale, ranging from white to purple, reflects the -log(P_{smr}) values. A darker purple color indicates greater statistical significance, whereas a white color represents lower significance. Tiles with accompanying numbers indicate that the associated SMR effect reaches the significant threshold of 5.5×10^{-3} (correcting for 9 tests), with the number being the estimated SMR effect size.

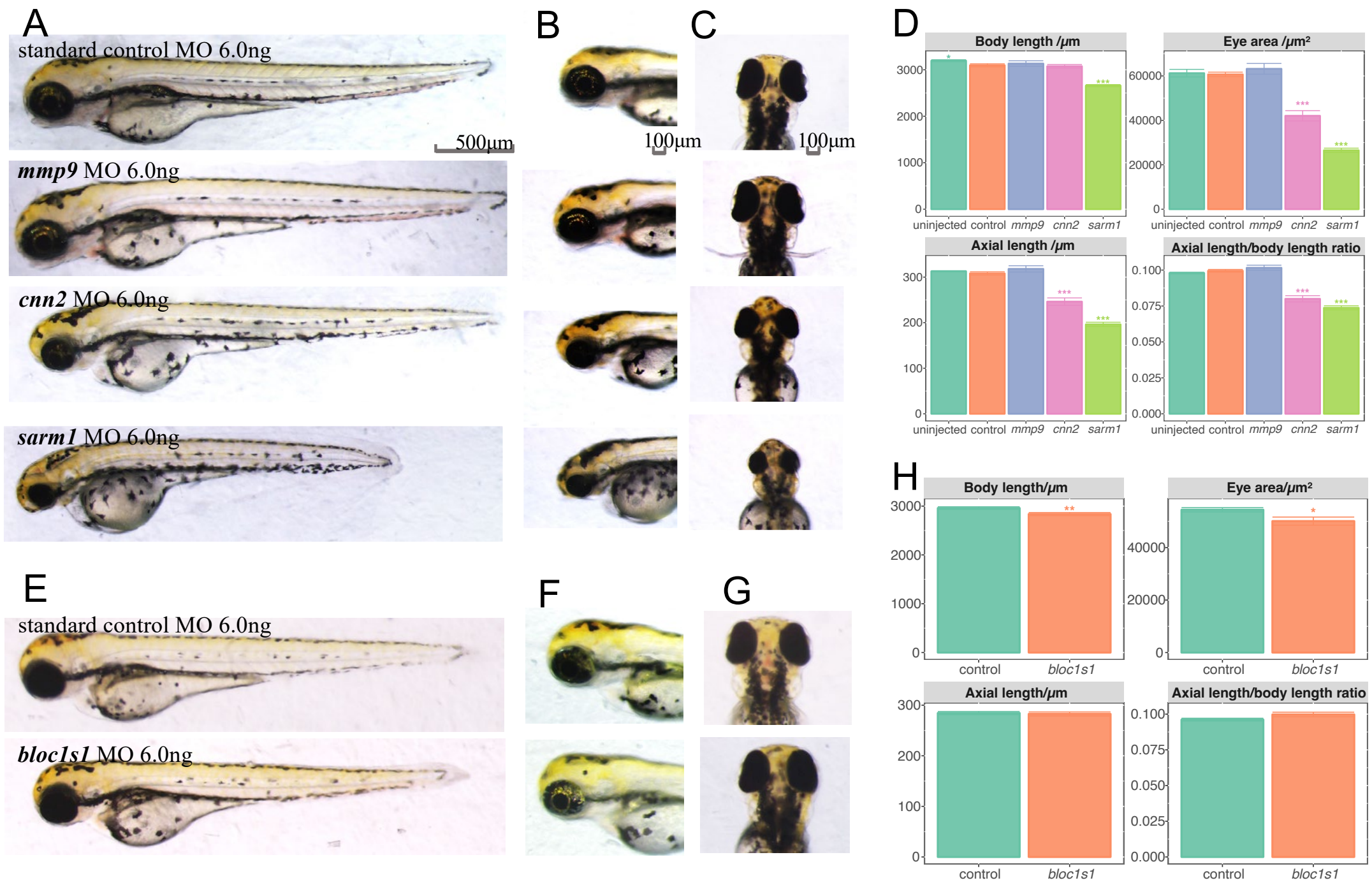

Fig. S2. Pre-experiments for morphology of *mmp9*-, *cnn2*-, *sarm1*-, and *bloc1s*-deficient zebrafish morphants. All embryos were injected with the corresponding MO at a dose of 6.0 ng ($n = 10$ for each group). (*A*, *E*) Lateral view of whole bodies. (*B*, *F*) Magnified lateral view of zebrafish eyeballs. (*C*, *G*) Magnified vertical view of zebrafish eyeballs. (*D*, *H*) Barplots of the eye area, axial length, and the ratio of axial length and body length, respectively. A significant reduction in axial length and eye area was observed in *cnn2*- and *sarm1*-deficient fish. Bar plots are shown as the mean ± s.e.m. Two-sided Student's t-test was performed between each group and the standard control. **P*<0.05, ***P*<0.01, ****P*<0.001.

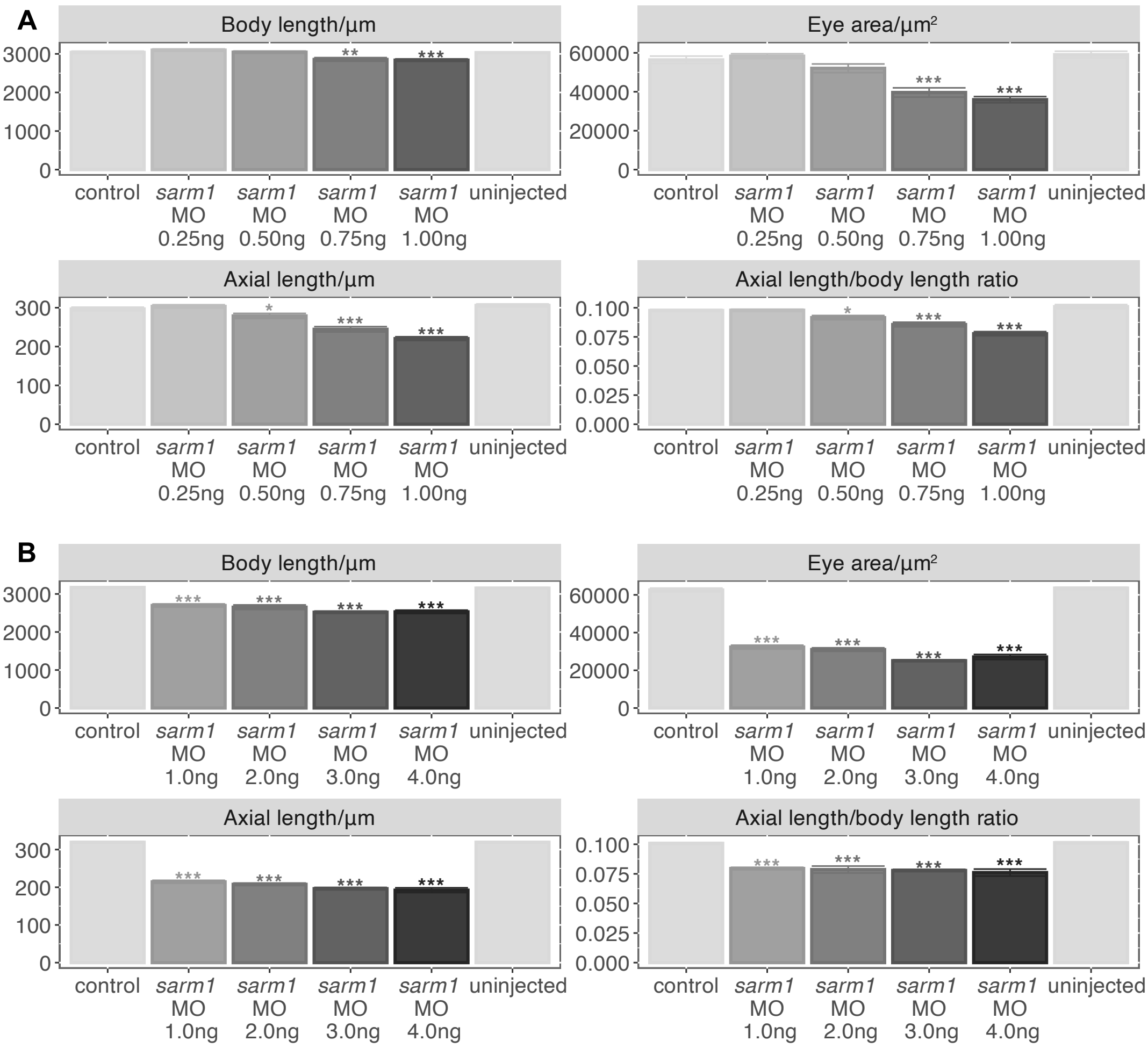

Fig. S3. Dose-dependence of ocular phenotypes for *sarm1*-deficient zebrafish morphants. (*A*, *B*) Bar plots showing eye area, axial length, and the ratio of axial length and body length across different MO doses. Decreased axial length and eye area occurred at a dose of 0.50 ng, and decreased body length occurred at a dose of 0.75 ng. The measurements decreased as the MO dose increased and then plateaued at and above 3.0 ng. Bar plots were shown as the mean ± s.e.m. Two-sided Student's t-test was performed between each group and the standard control. *P<0.05, **P<0.01, ***P<0.001.

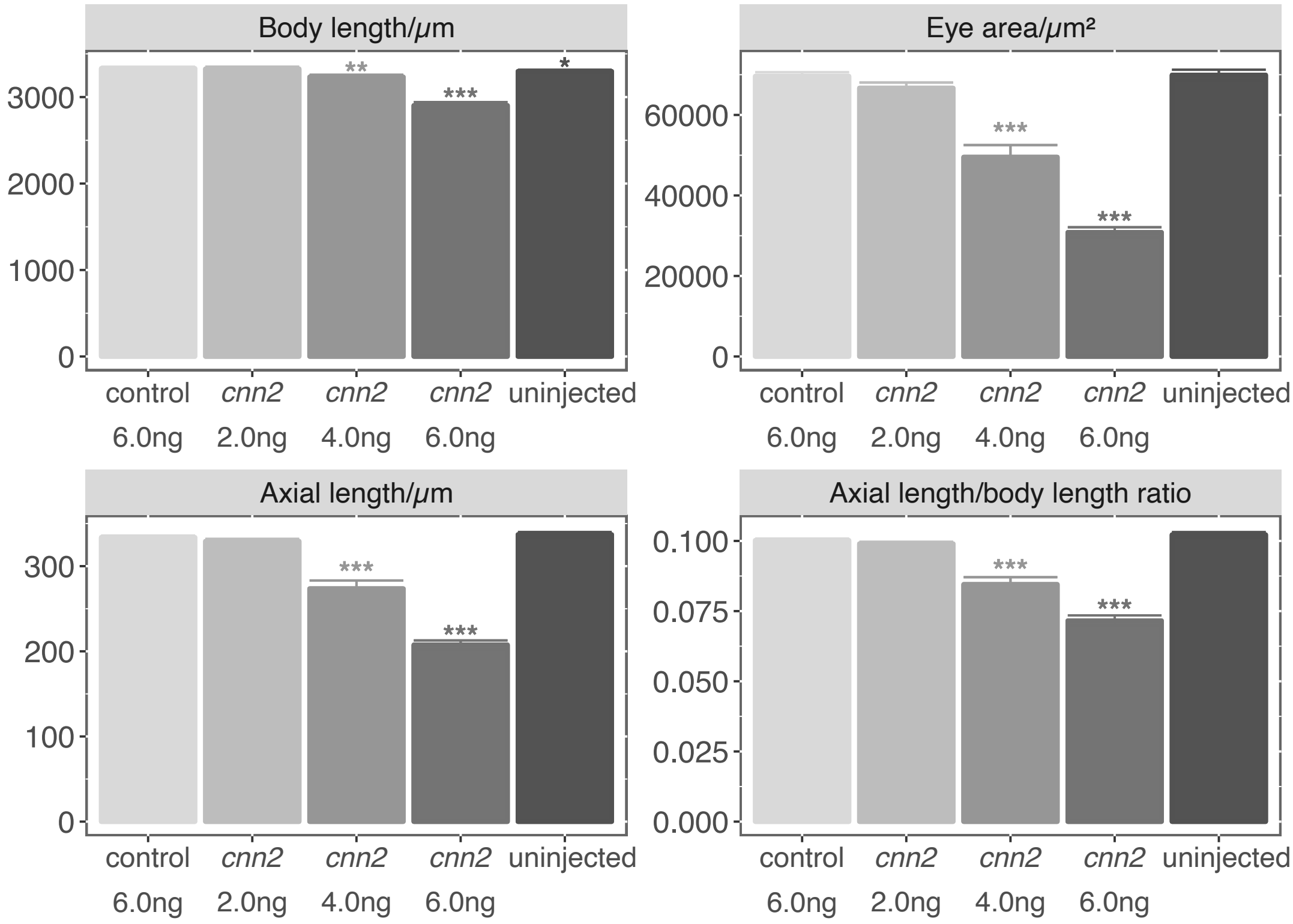

Fig. S4. Dose-dependence of ocular phenotypes for *cnn2*-deficient zebrafish morphants. Bar plots showing eye area, axial length, and the ratio of axial length and body length across different MO doses. The reductions in these measurements increased with MO dose over the 4.0–6.0 ng range. Bar plots were shown as the mean ± s.e.m. Two-sided Student's t-test was performed between each group and the standard control. *P<0.05, **P<0.01, ***P<0.001.

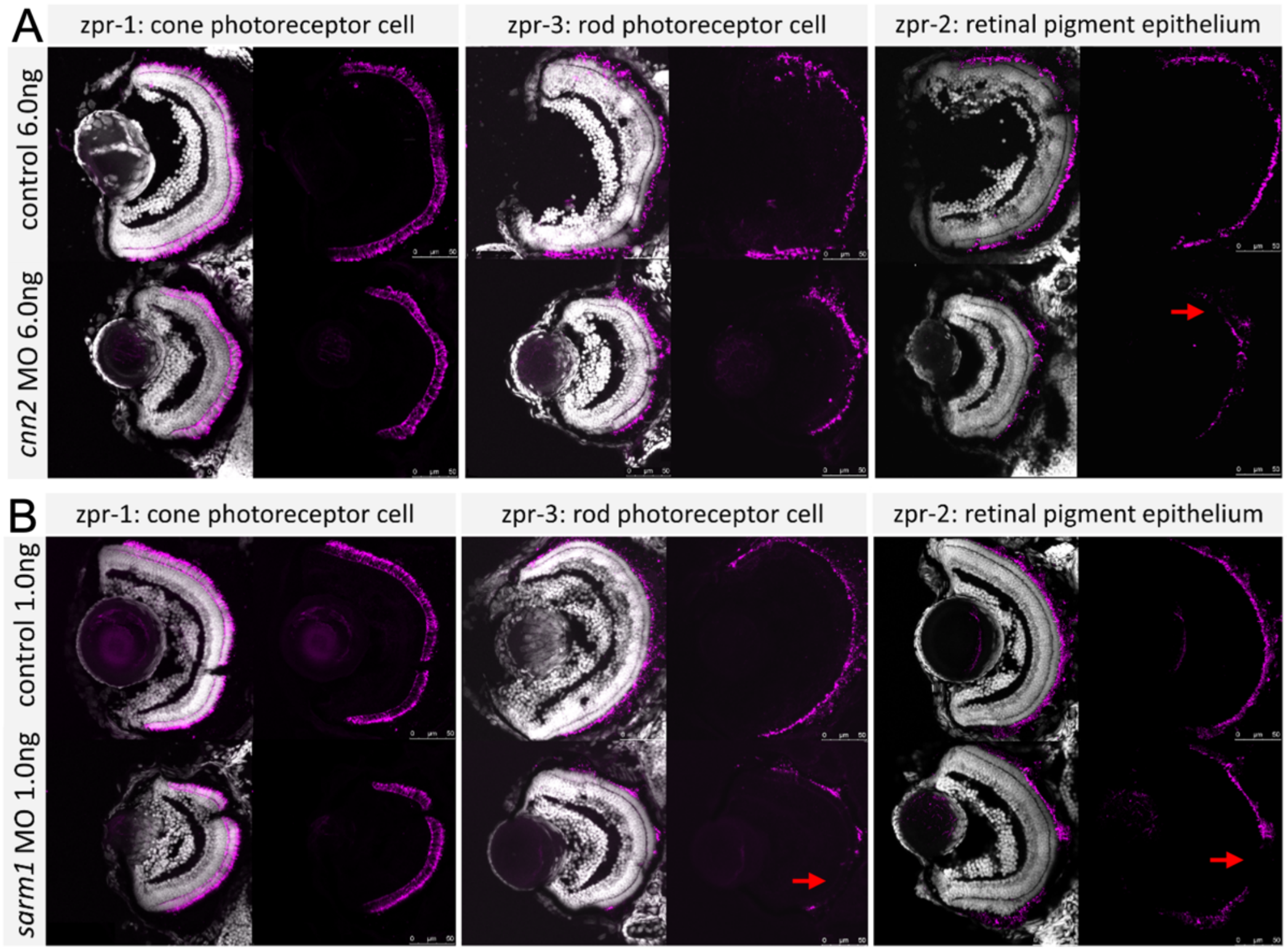

Fig. S5. Retinal architecture of *cnn2*- and *sarm1*-deficient morphants at 3 dpf. Nuclear layers were stained by DAPI in grey, and each group showed intact lamination. zpr-1, zpr-2 and zpr-3 were stained in purple for each column. (*A*) Signals of zpr-1 and zpr-3 in the *cnn2* MO 6.0 ng group were detected at relatively normal levels, but the signal of zpr-2 was much lower compared with the standard control. (*B*) Signals of zpr-3 and zpr-2 were markedly reduced in the outer layer of retina for the *sarm1* MO 1.0 ng group, and zpr-1 remained intact.

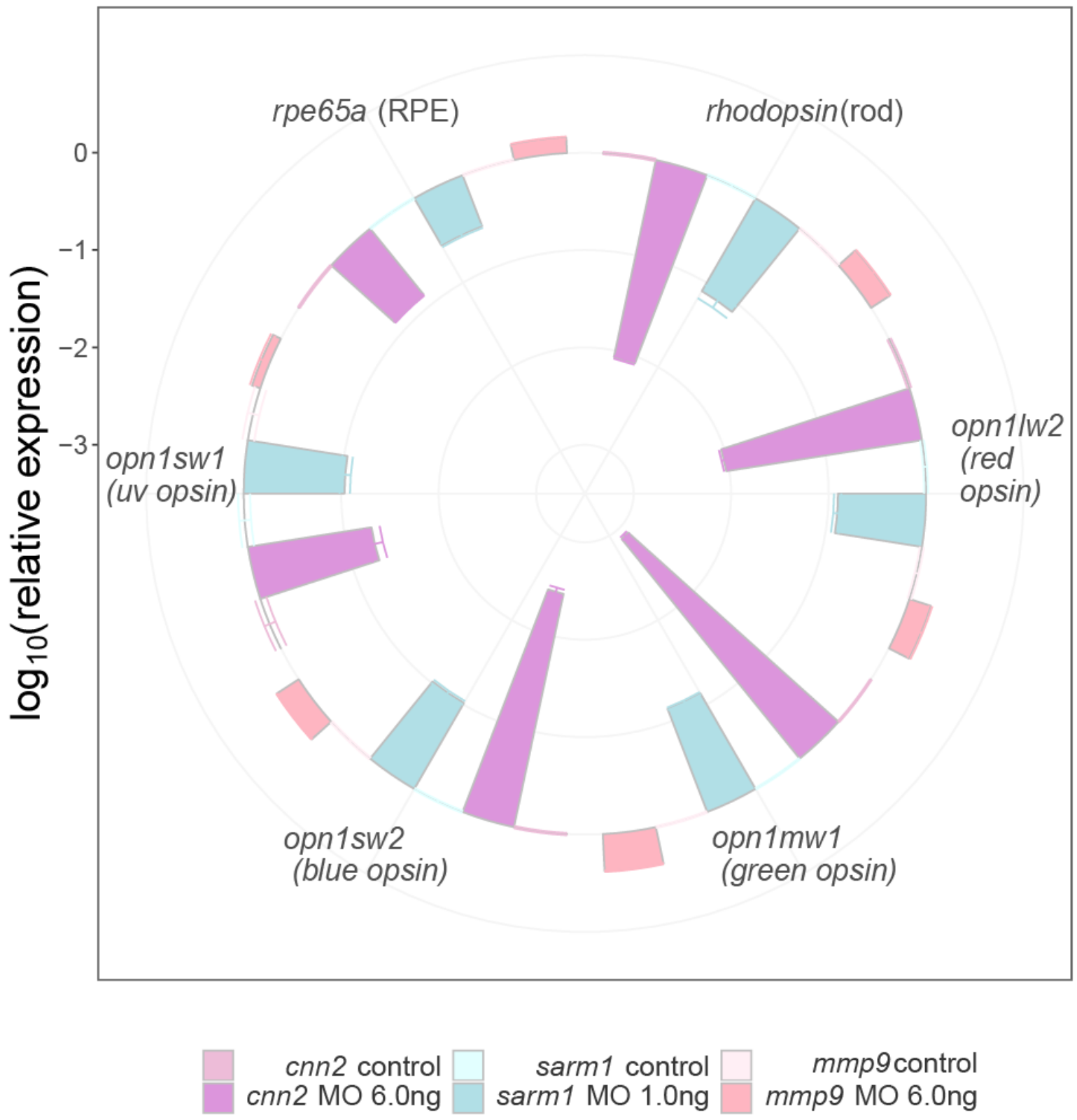

Fig. S6. Real-time qPCR of photoreceptor and RPE genes in the MO zebrafish oculus. The x axis represents vital photoreceptor and RPE genes, and the y axis represents the $log_{10}\left(relative\ expression\right)$. Centripetal bars indicate downregulation, and centrifugal bars indicate upregulation. The *cnn2*- and *sarm1*-MO groups revealed relatively low expression for all gene markers, whereas the *mmp9* group showed a slight increase. The bar plot is shown as the mean ± s.e.m.

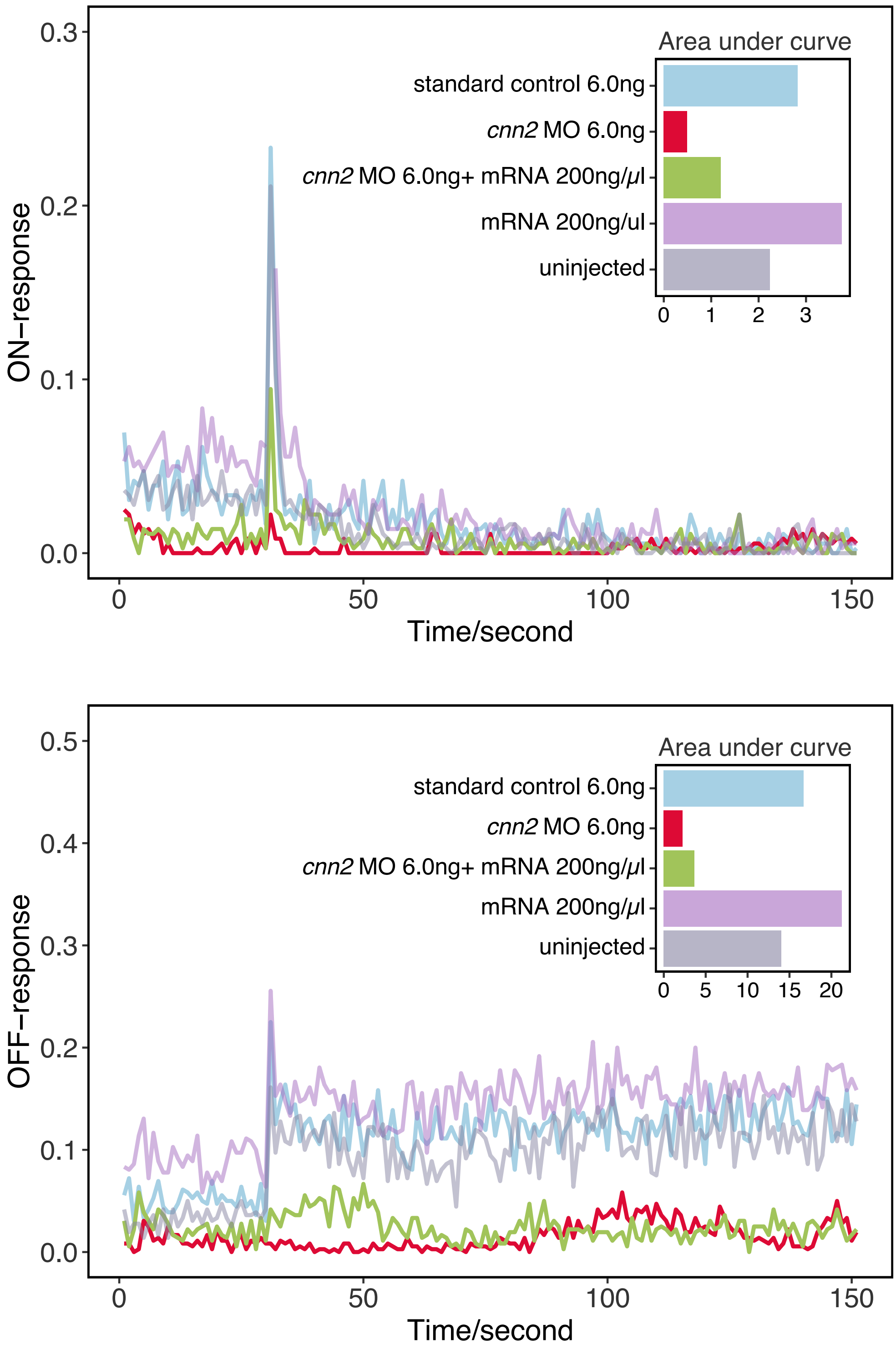

Fig. S7. Pre-experiment for visual function of *cnn2* in zebrafish larvae. VMR tests evaluating light-on and light-off responses. Functional recovery relied on a sufficient dose of injected mRNA. Partial rescue of visual function was observed after injection of 200 ng/μl *cnn2* mRNA.

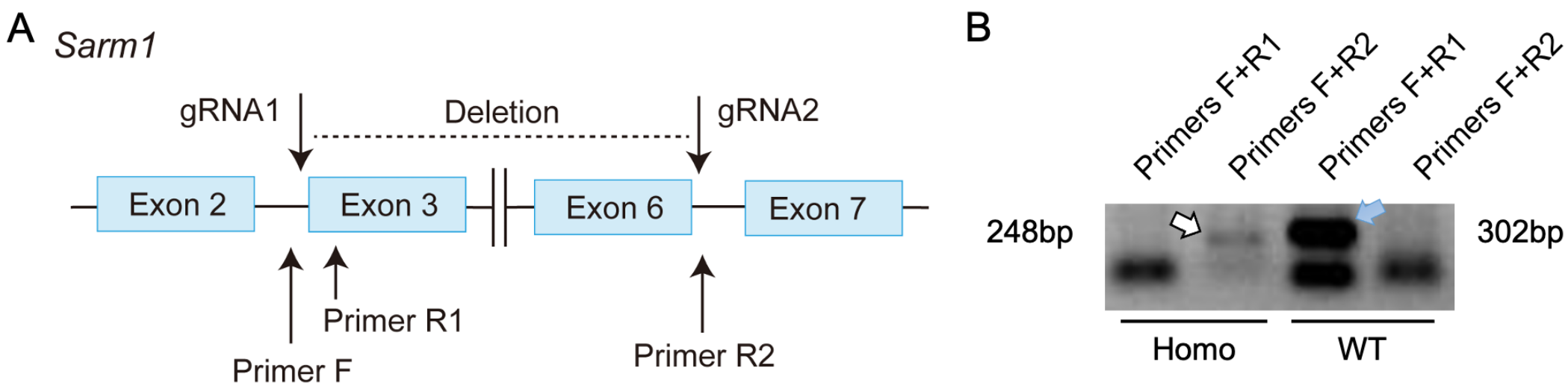

Fig. S8. Generation of *Sarm1* knockout mice. (*A*) Schematic representation of the *Sarm1* knockout strategy in mice. Exons 3 to 6 were targeted for knockout, and specific PCR primers were designed flanking the knockout region. Primer F, in combination with primer R1, enabled the detection of the non-knockout allele, while primer F, in combination with primer R2, enabled the detection of the knockout allele. (*B*) PCR results verifying the successful generation of *Sarm1* knockout mice. In wild-type mice, a 302 bp band was observed, whereas knockout mice displayed a 248 bp band. The white and blue arrows denote the knockout and wildtype bands, respectively. The bottom bands observed represent primer dimers formed during the PCR amplification process.

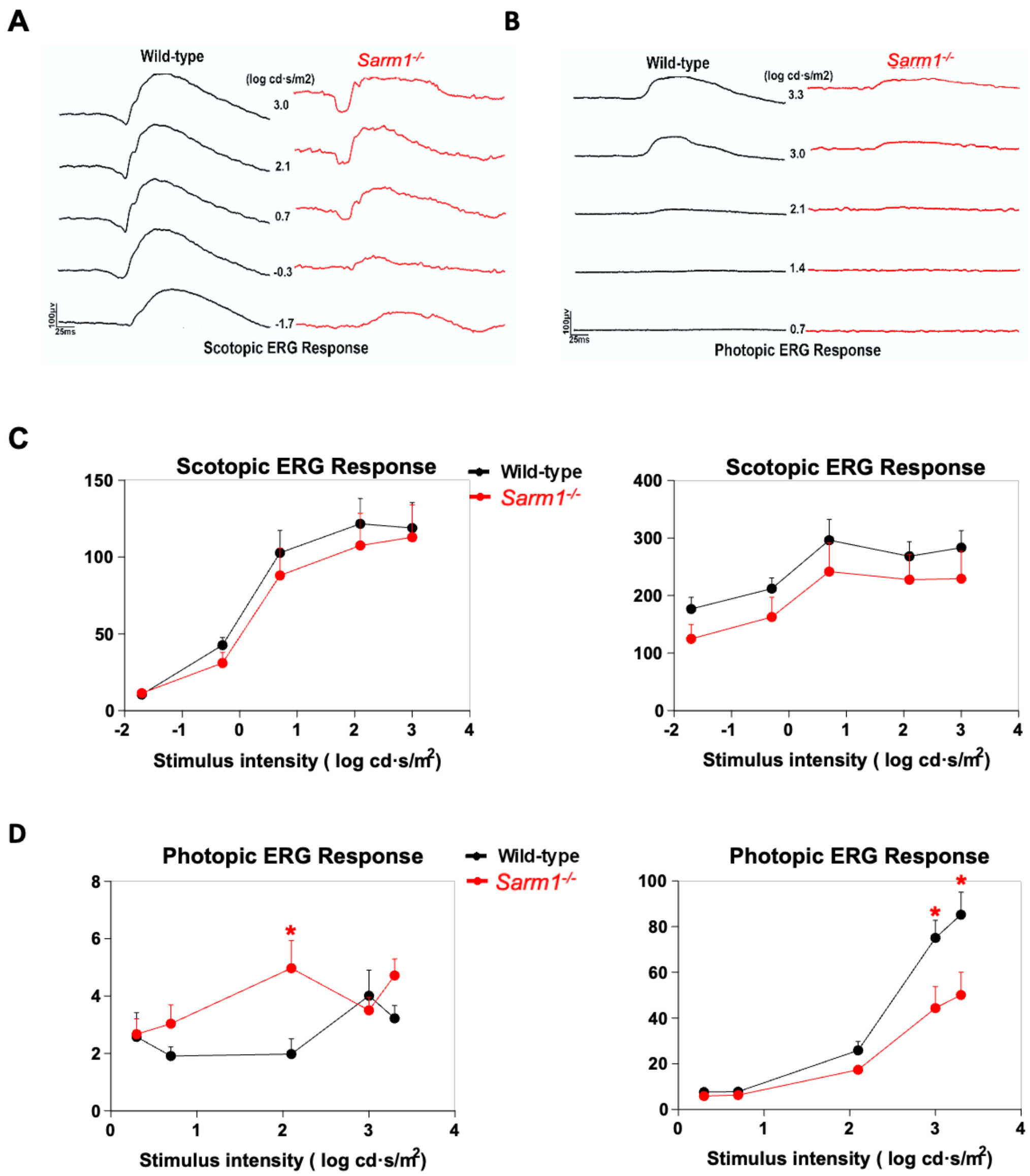

Fig. S9. ERG recordings in *Sarm1* knockout mice at 4.5 months of age. (*A*) Representative scotopic ERG recording curves. (*B*) Representative photopic ERG recording curves. (*C*) Quantitative measurements of scotopic ERGs. ERG recordings were stimulated at different intensities as indicated on the x-axis. (*D*) Quantitative measurements of photopic ERGs with stimuli at different intensities.

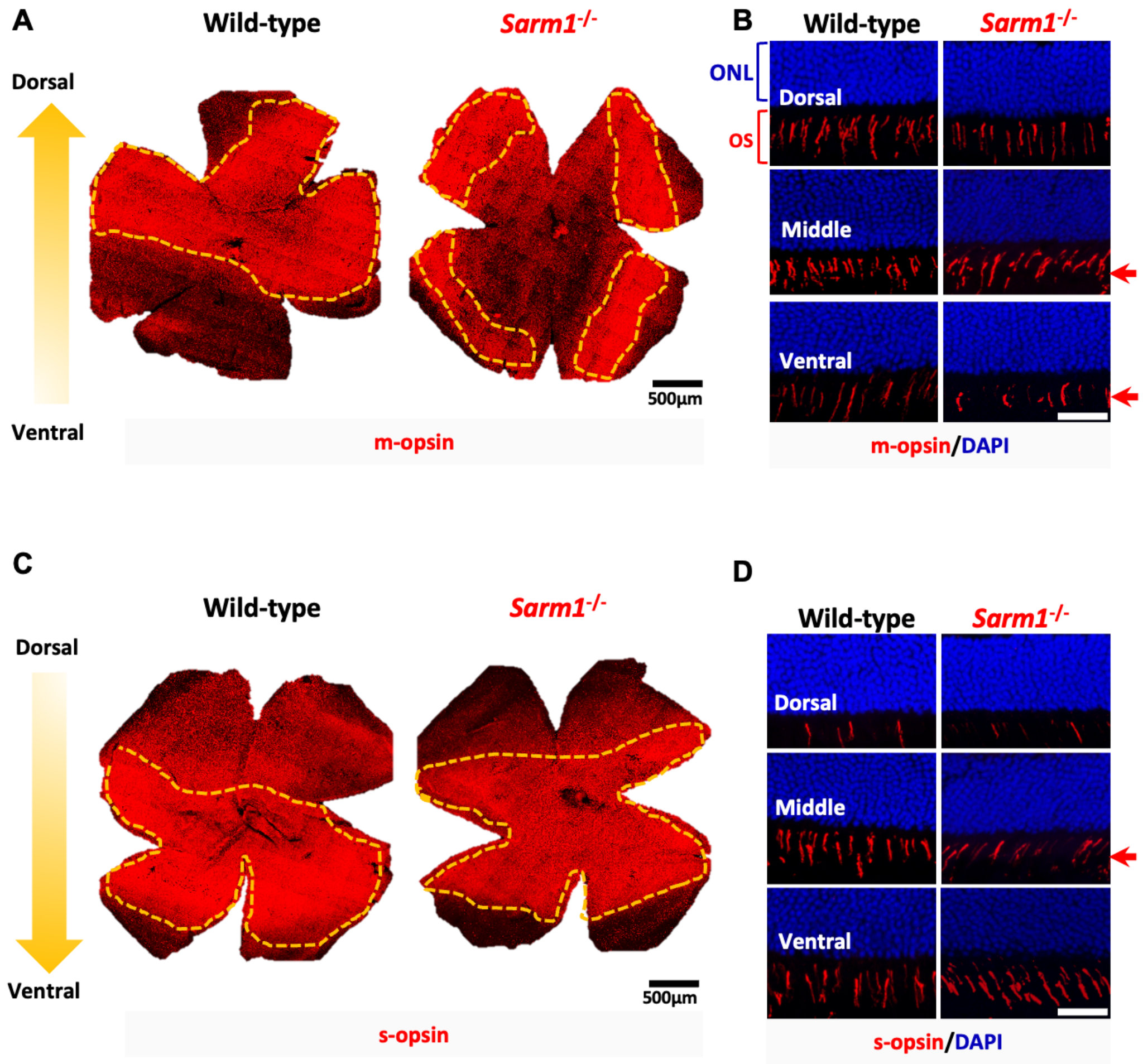

Fig. S10. Immunostaining of cone markers in the *sarm1* knockout mice at 4.5 months of age. (*A*) Immunofluorescence staining of M-opsin on retinal whole mounts for the control and *sarm1* KO mice. (*B*) Representative images depicting M-opsin staining in the dorsal, middle, and ventral regions, respectively. (*C*) Immunofluorescence staining of S-opsin on retinal whole mounts for the control and *sarm1* KO mice. (*D*) Representative images of S-opsin staining in the dorsal, middle, and ventral regions, respectively. Relatively sparse cone marker signals were observed in the *sarm1* KO mice, particularly in the middle region.

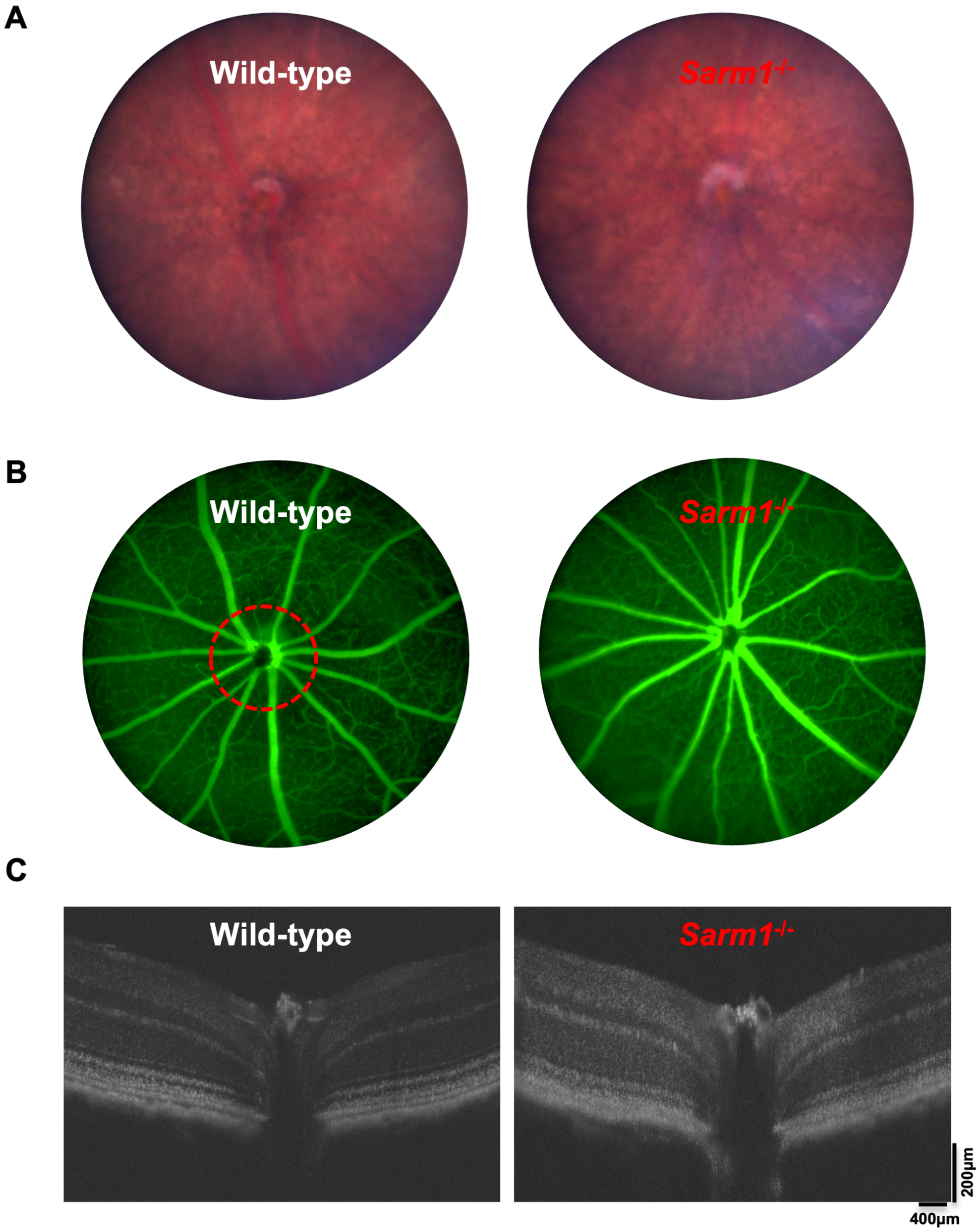

Fig. S11. Structural phenotypes of the retina in the *Sarm1* knockout mice at 4.5 months of age. (*A*) Color fundus photography comparing the retinal appearance of control and *Sarm1* KO mice. (*B*) Fundus fluorescent angiography images illustrating the vascular characteristics of control and *Sarm1* KO mice. (*C*) OCT images capturing the retinal layers in control and *Sarm1* KO mice. No discernible abnormalities were observed in the retinal structure of the *Sarm1* KO mice when compared to the control group.

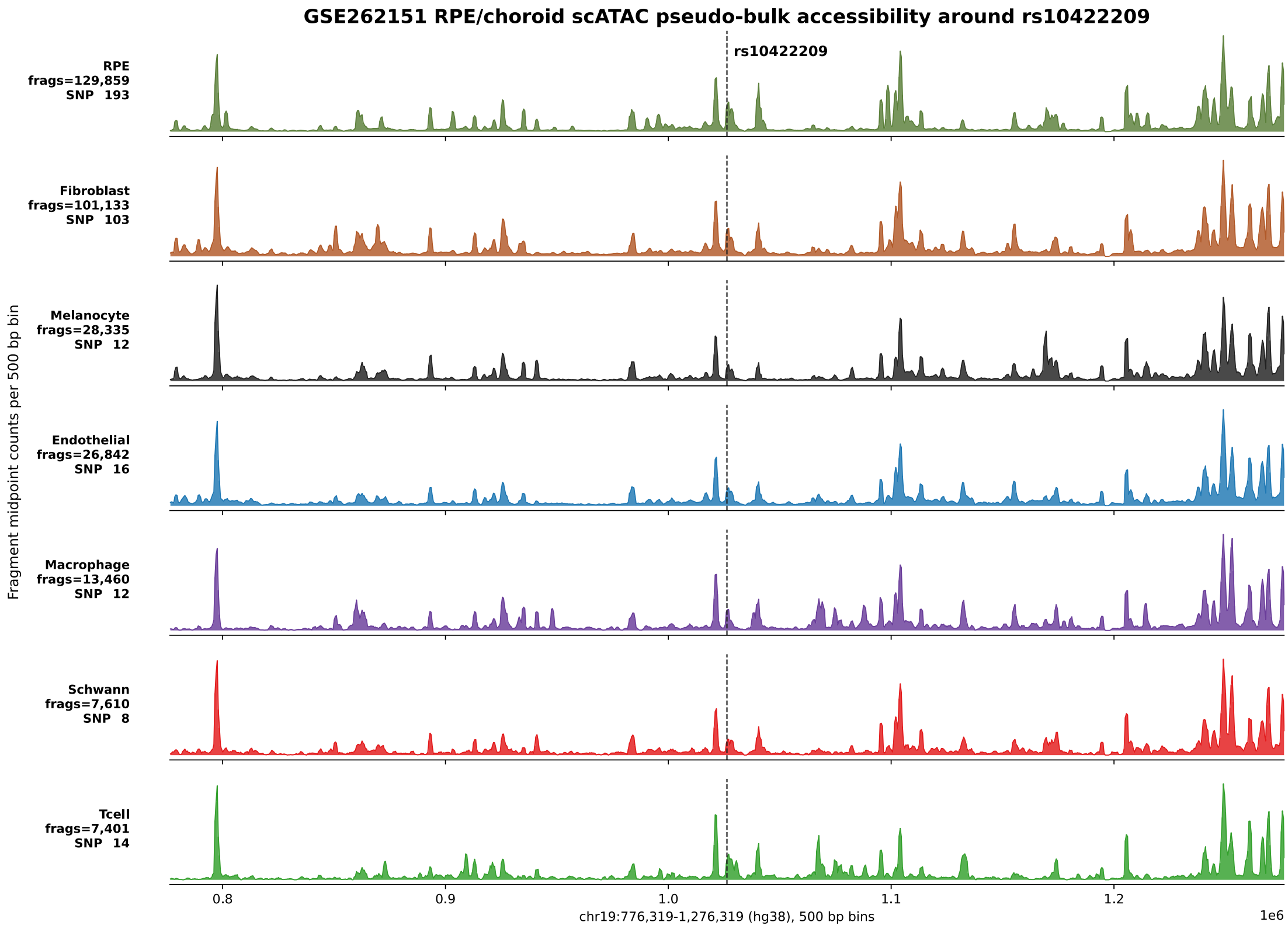

Fig. S12. Cell-type-specific chromatin accessibility around rs10422209 in human RPE/choroid tissue. Pseudo-bulk chromatin accessibility profiles were generated from the GSE262151 single-cell ATAC-seq dataset by aggregating fragments within each annotated cell type, including RPE, fibroblast, melanocyte, endothelial, macrophage, Schwann, and T cells. Accessibility is shown as the number of ATAC-seq fragment midpoints per 500-bp genomic bin across the chr19:776,319–1,276,319 region (hg38). The vertical dashed line indicates the genomic position of rs10422209 (a fine-mapped variant in the *CNN2* locus). A local accessibility signal was observed at or near rs10422209, with the most prominent signals detected in RPE cells. The total number of fragments included in each pseudo-bulk profile and the fragment count for the SNP-containing bin are indicated to the left of each track.

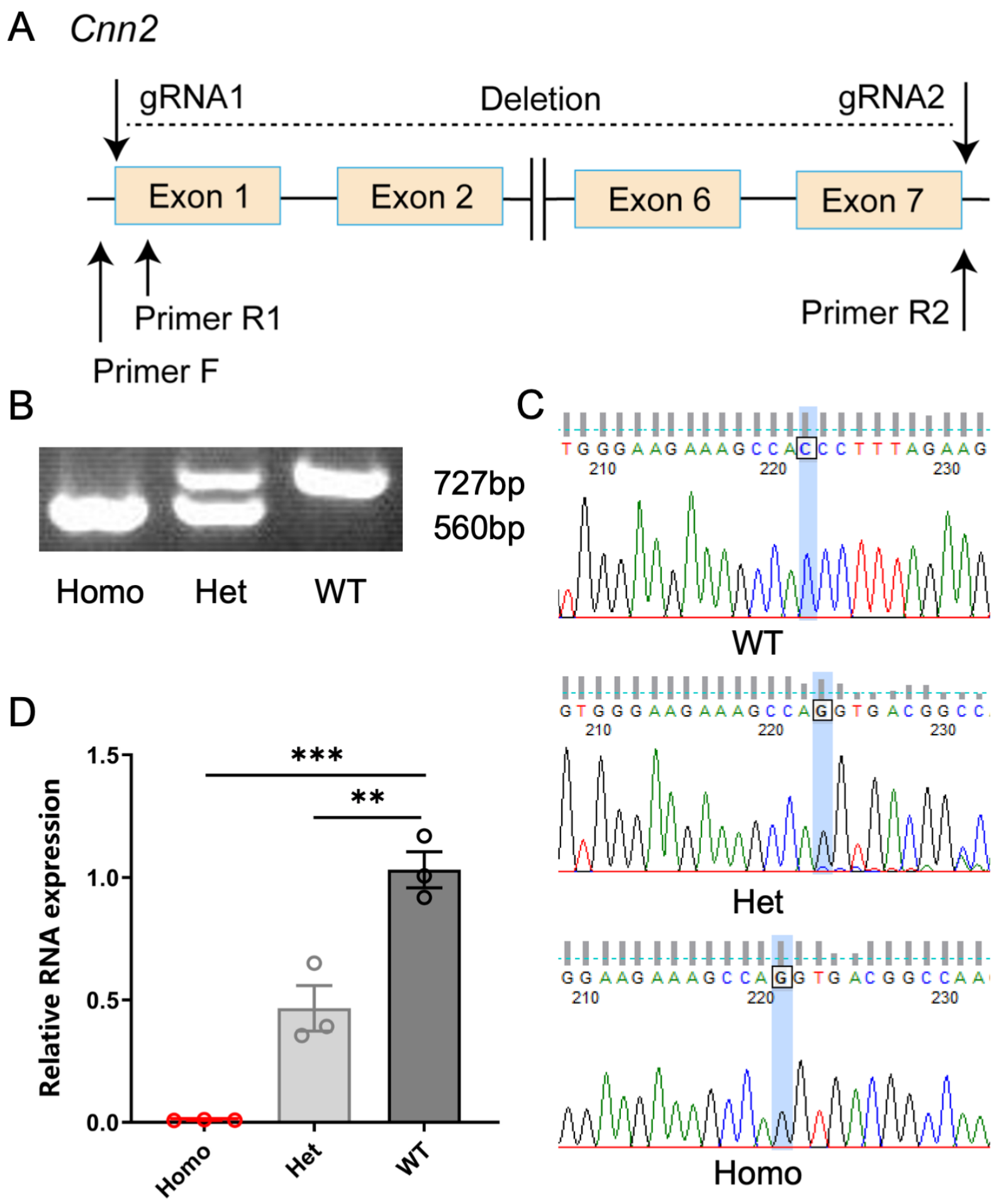

Fig. S13. Generation and confirmation of *Cnn2*-knockout mice. (*A*) Schematic representation illustrating the generation of *Cnn2*-knockout mice using the CRISPR/Cas9 strategy. (*B*, *C*) Confirmation of the knockout success by Sanger sequencing analysis of germline DNA. (*D*) Quantitative RT-PCR analysis demonstrating the relative *Cnn2* gene expression levels of homozygous (*Cnn2* $^{-/-}$), heterozygous (*Cnn2* $^{+/-}$), and wild-type mice. The bar plot represents the mean ± s.e.m. with a sample size of $n = 3$ for the wildtype (WT) group, heterozygous (Het) and homozygous (Homo) groups, respectively. Statistical analysis was performed using a one-way ANOVA followed by Bonferroni post-hoc tests for pairwise comparisons between each group and the wild-type group, **P*<0.05, ***P*<0.01, ****P*<0.001.

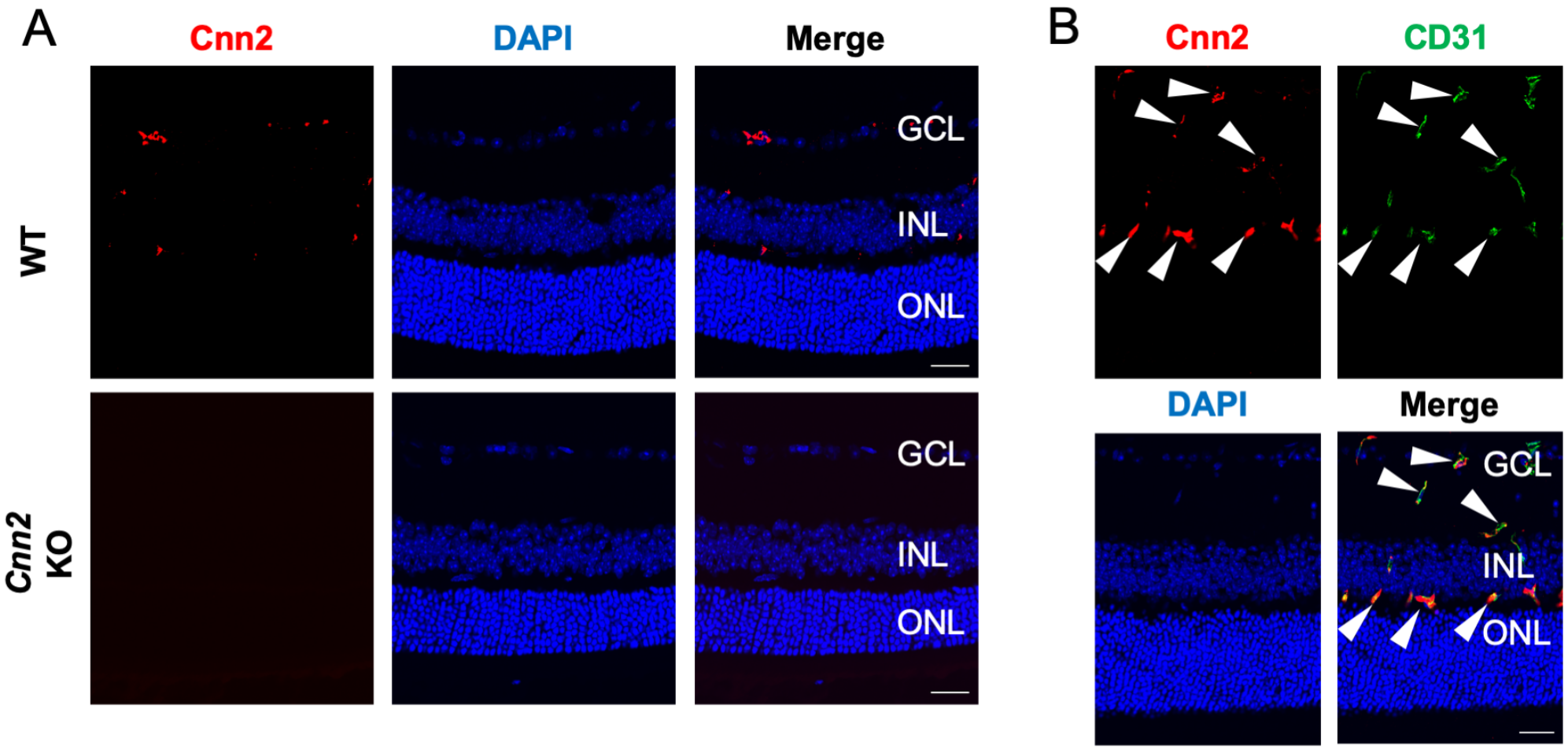

Fig. S14. Representative immunostaining images of *Cnn2*-Knockout mice. (*A*) Confocal image showing the expression levels of Cnn2. Cnn2 was expressed in the GCL, INL, and ONL. (*B*) Confocal images showing the co-localization of Cnn2 and CD31 in WT mouse retinas. White arrows indicate the co-localization of Cnn2 and CD31. The figures are magnified at 40×, and the scale bar of figures is 20 μm. GCL, ganglion cell layer; INL, inner nuclear layer; ONL, outer nuclear layer.

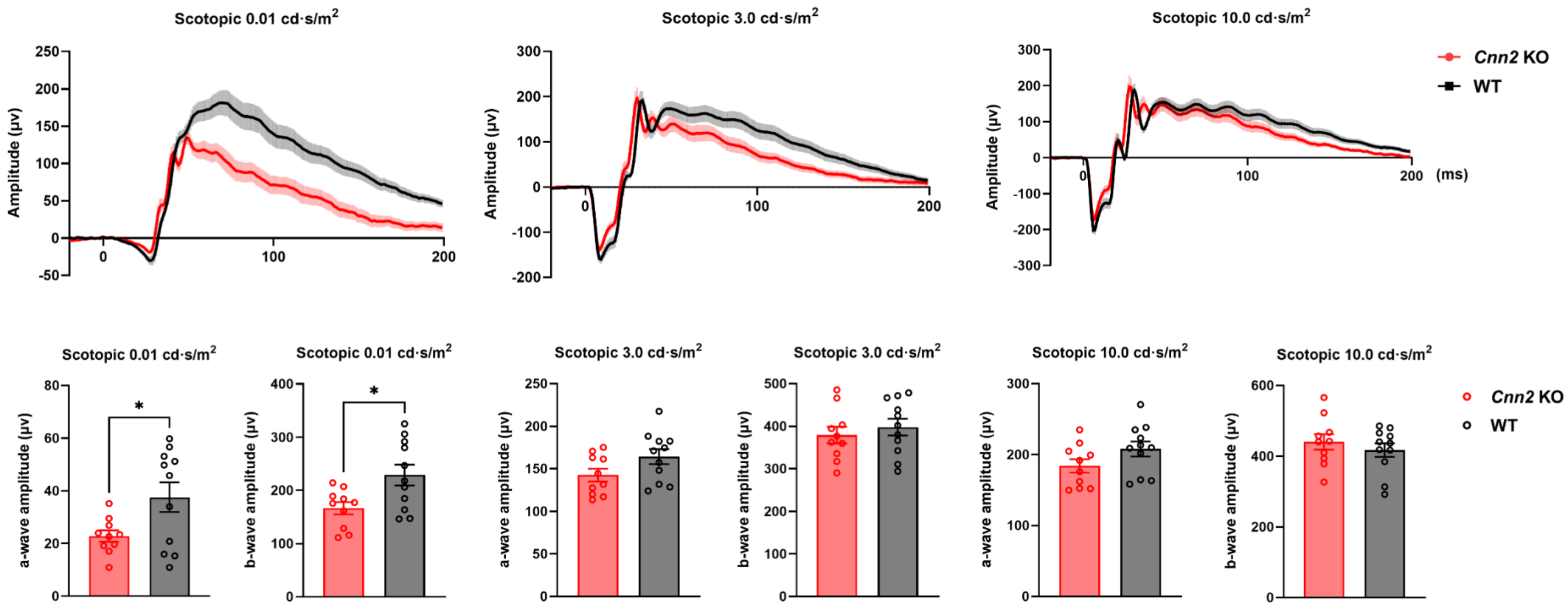

Fig. S15. Scotopic ERG waves and quantitative measurements of *Cnn2*-knockout mice. The bar plots in all panels represent the mean ± s.e.m. with a sample size of $n = 11$ for the wildtype (WT) group and $n = 10$ for the homozygous (*Cnn2* KO) group. Statistical analysis was performed using a two-sample t-test between each knockout group and the standard control, *P<0.05, **P<0.01, ***P<0.001.

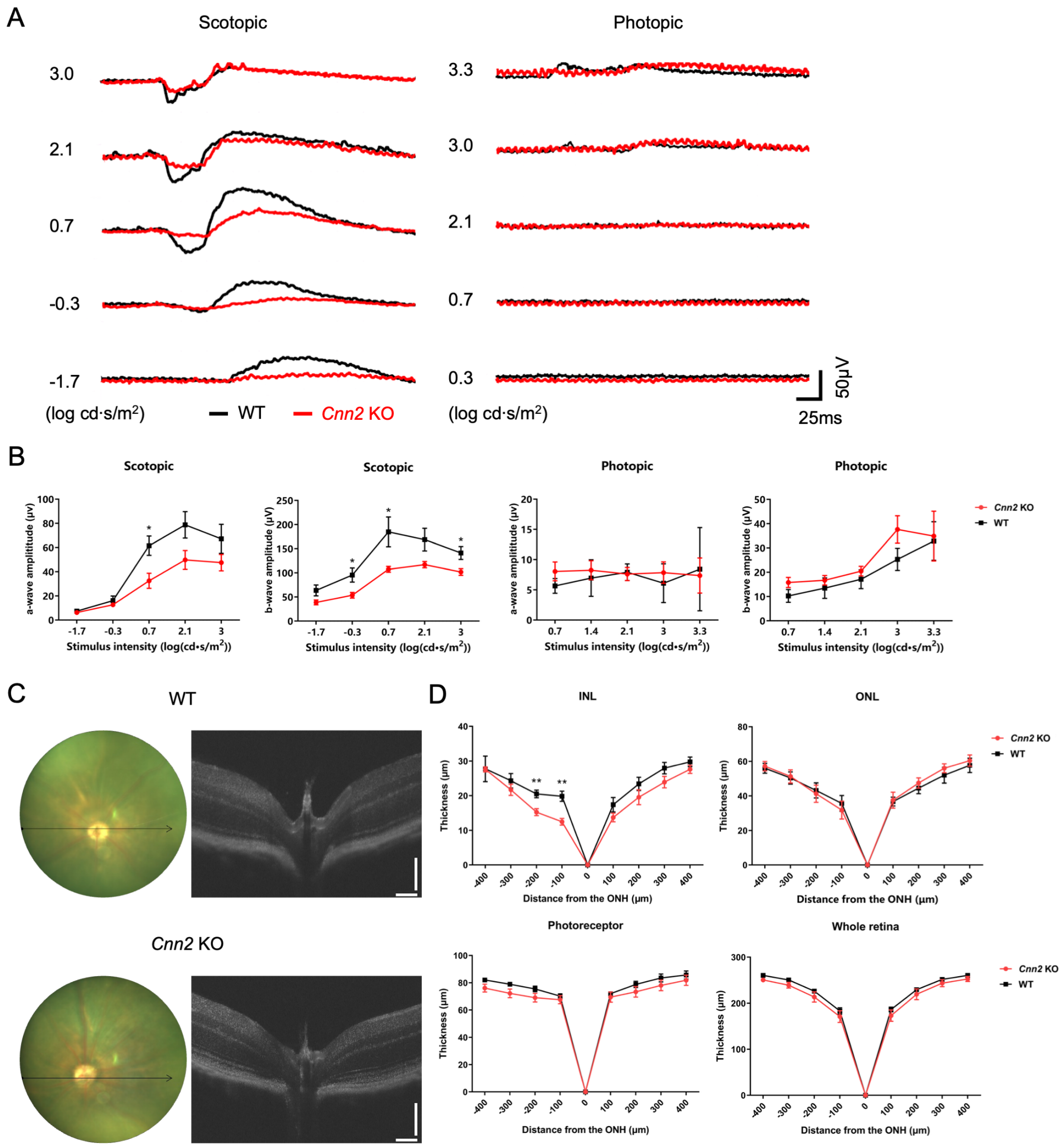

Fig. S16. Phenotypes of *Cnn2* knockout mice at 1 months of age. (*A*) Representative scotopic and photopic ERG recording curves. (*B*) Quantitative measurements of scotopic and photopic ERGs. ERG recordings were stimulated at different intensities and obtained from C57BL/6J and *Cnn2* knockout mice. $n = 4$ (WT) and $n = 4$ (*Cnn2* KO). (*C*) Fundus photography (FP) and Spectral-Domain Optical Coherence Tomography (SD-OCT) of *Cnn2* knockout mice. The horizontal scale bar is 100 μm and vertical scale bar is 100μm. (*D*) Quantitative measurements of OCT results. The thickness of the inner nuclear layer (INL), outer nuclear layer (ONL), photoreceptor layer and total retina were shown in the panel. $n = 4$ (WT) and $n = 5$ (*Cnn2* KO). Bar plots of all panels were shown as the mean ± s.e.m. A two-sample t-test was performed to compare each group with the standard control, **P*<0.05, ***P*<0.01, ****P*<0.001.

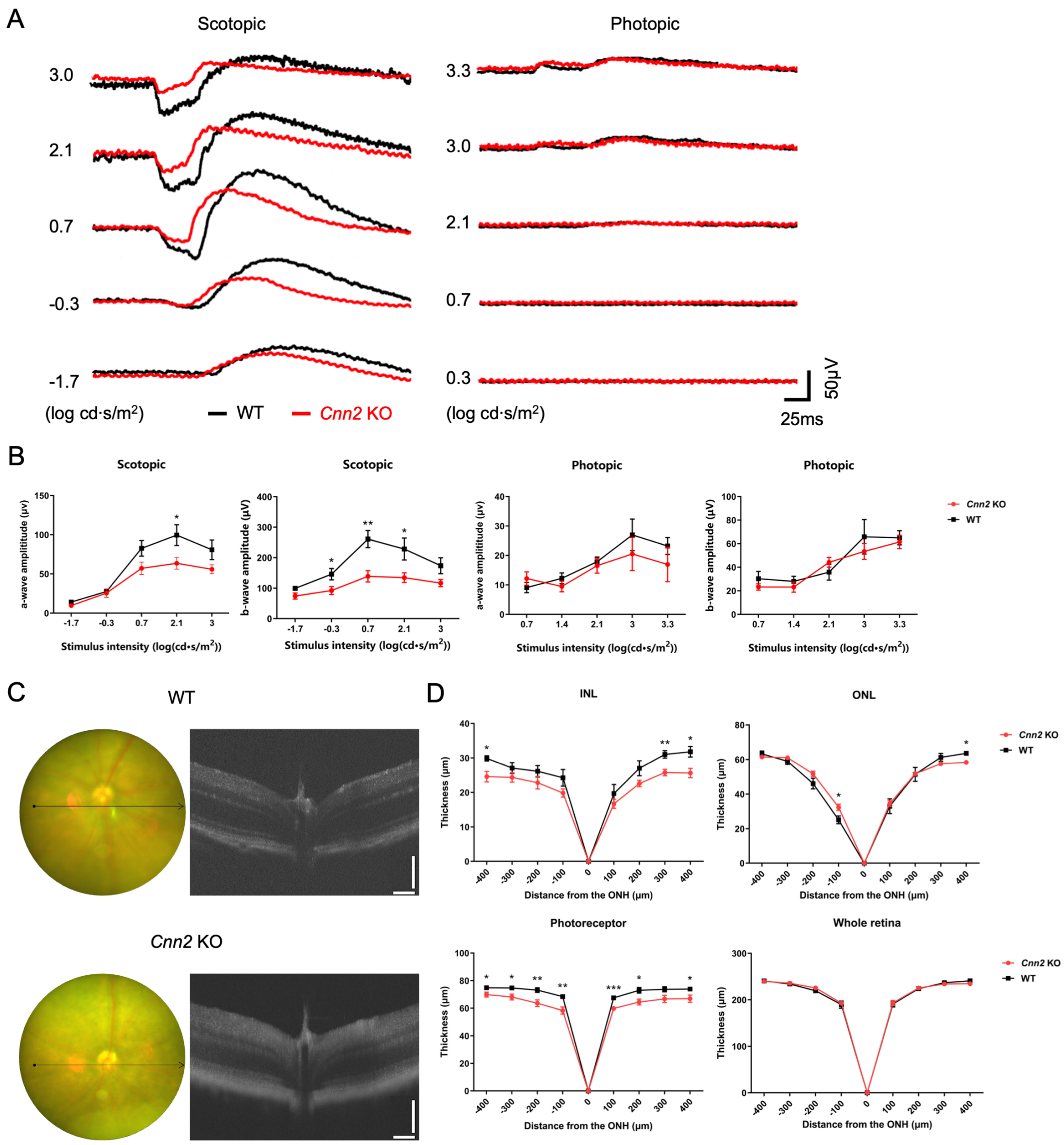

Fig. S17. Phenotypes of *Cnn2* knockout mice at 5 months of age. (*A*) Representative scotopic and photopic ERG recording curves. (*B*) Quantitative measurements of scotopic and photopic ERGs. ERG recordings were stimulated at different intensities and obtained from C57BL/6J and *Cnn2* knockout mice. $n = 4$ (WT) and $n = 6$ (*Cnn2* KO). (*C*) Fundus photography (FP) and Spectral-Domain Optical Coherence Tomography (SD-OCT) of *Cnn2* knockout mice. The horizontal scale bar is 100μm and vertical scale bar is 100μm. (*D*) Quantitative measurements of OCT results. The thickness of the inner nuclear layer (INL), outer nuclear layer (ONL), photoreceptor layer and total retina were shown in the panel. $n = 6$ (WT) and $n = 5$ (*Cnn2* KO). Bar plots of all panels were shown as the mean ± s.e.m. A two-sample t-test was performed to compare each group with the standard control, *P<0.05, **P<0.01, ***P<0.001.

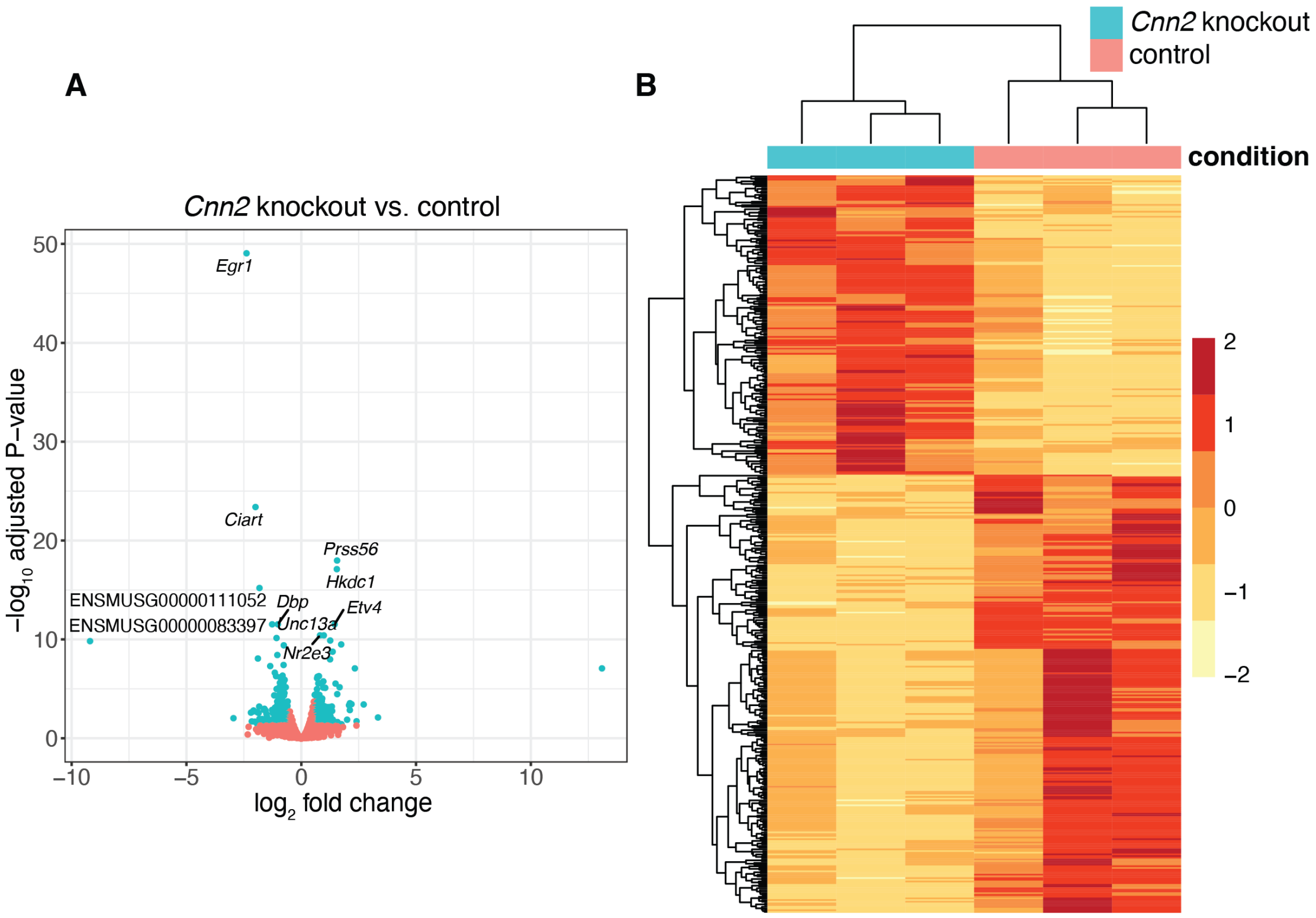

Fig. S18. Differentially expressed genes identified in the comparison between *Cnn2*-knockout mice and normal controls. (*A*) The volcano plot displays the log fold change on the x-axis and the negative logarithm of the *P*-value ($-\log_{10}$(*P*-value)) on the y-axis, based on RNA-seq results. Blue dots represent the differentially expressed genes (DEGs) with an FDR *P* value < 0.05 and a fold change greater than 1.5. (*B*) Heatmap depicting the expression profiles of DEGs across samples. Each row corresponds to a specific DEG, while each column represents a sample. The expression levels of the DEGs are visualized using a legend color scale.

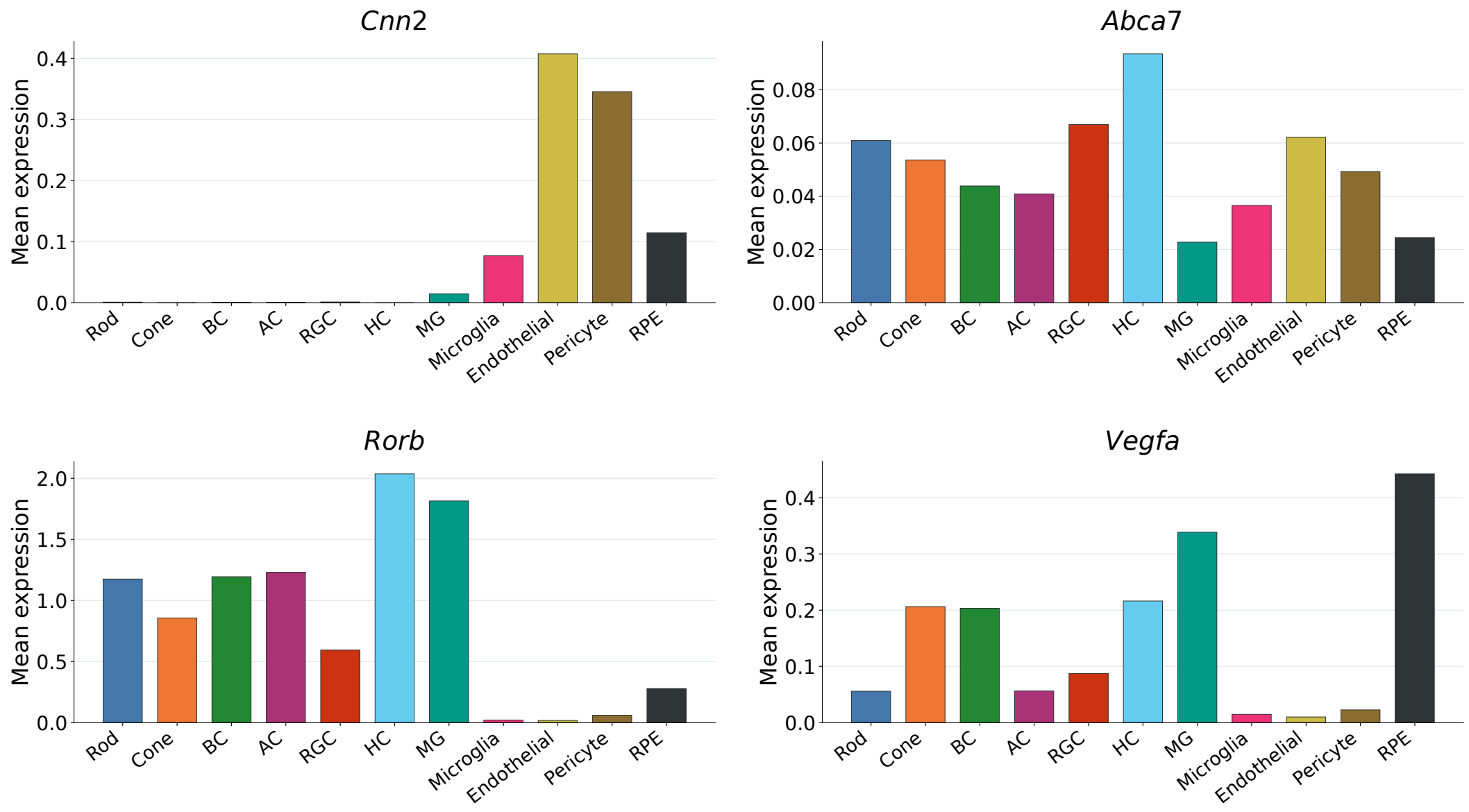

Fig. S19. Pseudo-bulk expression of selected genes across major mouse retinal cell classes. Bar plots show mean normalized single-cell expression of *Cnn2*, *Abca7*, *Rorb*, and *Vegfa* across major retinal cell classes from the Mouse Retina Cell Atlas of Li et al. (*iScience* 2024), accessed via the UCSC Cell Browser. *Abca7*, *Rorb*, and *Vegfa* were differentially expressed in *Cnn2*-knockout retinas and correspond to genes located at genome-wide significant AMD-associated loci. *Cnn2* expression is enriched in endothelial cells and pericytes, with lower expression in retinal pigment epithelium (RPE). *Abca7* is broadly detected across retinal cell classes, with relatively higher expression in horizontal cells and vascular-associated populations. *Rorb* is most highly expressed in horizontal cells and Müller glia, consistent with retinal neuronal/glial subtype specificity. *Vegfa* shows strongest expression in RPE and Müller glia, with additional expression in cones, bipolar cells, and horizontal cells.

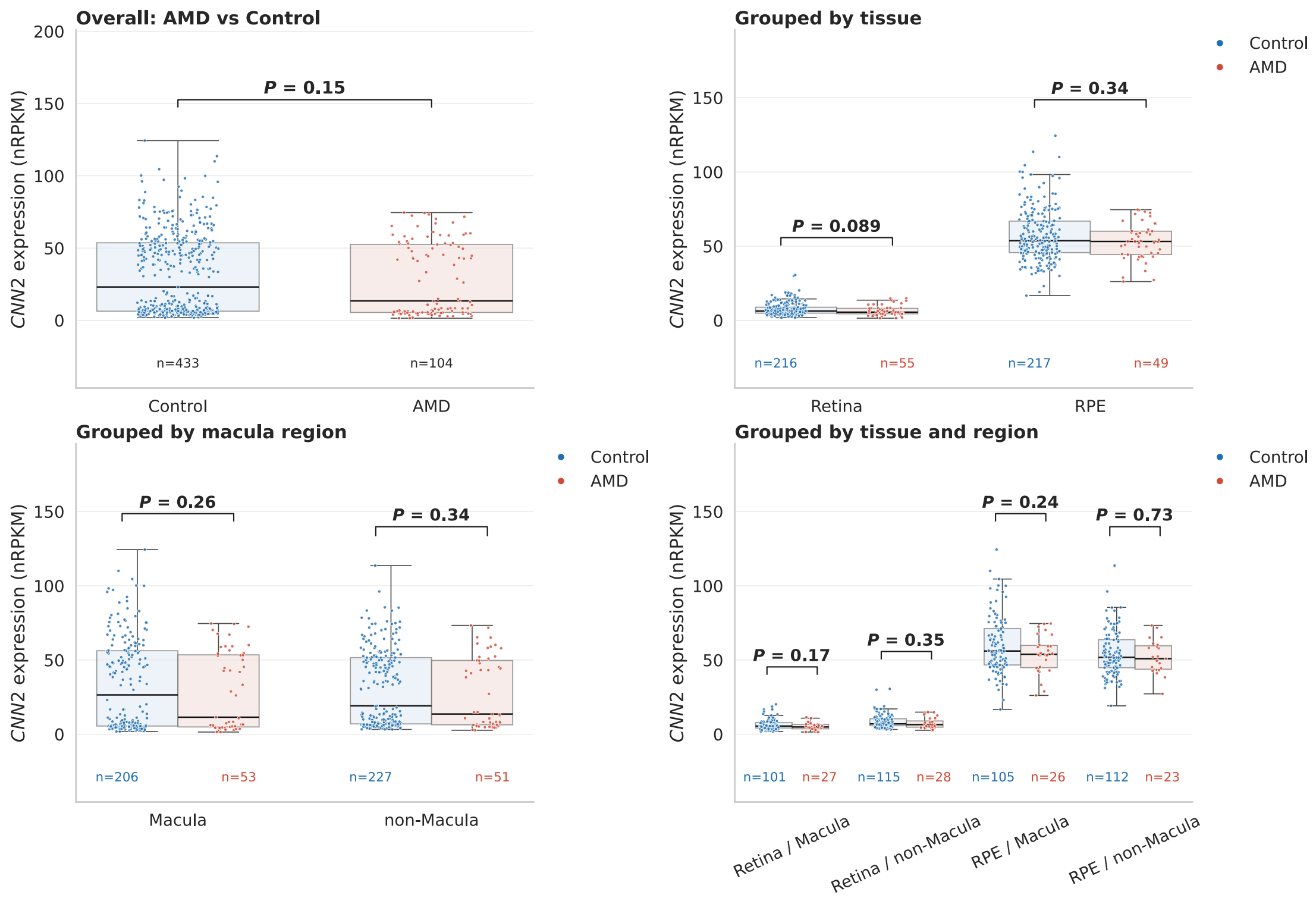

Fig. S20. *CNN2* expression according to AMD status, ocular tissue, and retinal region in the GSE135092 dataset. *CNN2* expression, measured as normalized reads per kilobase per million mapped reads (nRPKM), was compared between samples from donors with age-related macular degeneration (AMD) and control donors. Expression levels are shown for all samples combined (upper left), separately in retina and retinal pigment epithelium (RPE) tissues (upper right), by macular and non-macular region irrespective of tissue type (lower left), and after stratification by both tissue and retinal region (lower right). Each point represents an individual sample; blue and red points denote control and AMD samples, respectively. Box plots show the median and interquartile range, with whiskers indicating the distribution beyond the quartiles according to the standard 1.5 × interquartile-range convention. Sample sizes are indicated below each group. *P* values shown above the brackets were calculated using two-sided Mann–Whitney U tests comparing AMD and control samples within each indicated group.

Tables

Table S1. Functional annotations and reported disease relevance of nine prioritized candidate genes for AMD.

Gene Name	Functional annotation and reported disease relevance
ABCA7	ATP-binding cassette subfamily A member 7. The mRNA has a dominant expression in myelo-lymphatic tissues (17) and microglia in brain (18). It was suggested to play a role in macrophage transmembrane lipid transport1. Variants and epigenetic markers in *ABCA7* have been reported significant association with Alzheimer's disease (AD) (19), also an age-related disease. Knockout of *Abca7* showed no obvious phenotypic abnormalities but with serum lipid alternation in young mice (20).
BLOC1S1	Biogenesis of lysosome-related organelles complex 1 subunit 1. Mutation in the complexes results in Hermansky-Pudlak Syndrome, characterized by decreased pigmentation and lysosomal accumulation of ceroid lipofuscin, with also impaired vision (21).
C17orf90	Also called *OXLD1*, Oxidoreductase Like Domain Containing 1. Relevant literature is unavailable.
CNN2	Calponin 2 is expressed in a broader range of tissues and a significant level in macrophages. Deletion of *Cnn2* could accelerate macrophage migration and phagocytosis thus hinder the progress of atherosclerosis, a vascular inflammatory disease (22). On the other hand, it is also demonstrated that *cnn2* MO zebrafish had cardiovascular defects (23). Besides, alike *ABCA7*, *CNN2* resides in rs4147929 LD block, which is AD associated loci (24).
MMP9	Matrix metallopeptidase 9, is specific to wet AMD among all AMD-associated variants (4). In ophthalmology, *MMP9* participates in extracellular matrix remodeling and microvascular permeability during ocular angiogenesis in RPE and retinal microvascular endothelial cells (25). It also acts as a bio-marker to identify inflammatory dry eye and ocular surface diseases (26). Systemic pathological processes, like immunological diseases (27), cancer and its metastasis (28), cardiovascular (29) were found to associate *MMP9*.
PILRB	Paired immunoglobulin-like type 2 receptor beta, an activating immune receptor, distributes broadly across tissues and has a relative high expression in microglia (30). Genetic variants in *PILRB* drive not only the association susceptibility of neurodegenerative diseases, such as AD, Parkinson's disease (31), but also altered expression of *PILRB*, where the expression was lower in AD cases compared with controls (24). Additionally, rs61735533, labelling *PILRB* in the study, plays a larger role in European population (where it has a risk allele frequency of 18%-20%) than it does in Asian population (where is has a risk allele frequency of 3% in East Asian and 12% in South Asian), which is consistent with the prevalence difference of AMD in those population.
RDH5	Retinol Dehydrogenase 5. *RDH5* is one of causal genes of fundus albipunctatus, characterized with yellow and white lesion at RPE, with or without cone dystrophy (32). Macular cone density was detected lower for fundus albipunctatus patients with *RDH5* mutations (33).
SARM1	Sterile Alpha and TIR Motif Containing 1, a negative regulator of the Toll-like receptor signaling pathway in innate immunity and predominantly expressed in neurons (34). Knockdown of *Sarm1* negatively influences neuronal development and synaptic function (34, 35). It is also demonstrated loss-of-function of *Sarm1* delays degeneration of injured axons by preventing ATP depletion (36, 37).
TMEM199	Transmembrane Protein 199. Deficiency of *TMEM199* leads to congenital disorders of glycosylation with hypercholesterolemia because of Golgi homeostasis disruption (38). It is also involved in intracellular iron homeostasis required for endolysosomal acidification and lysosomal degradation (39).

Table S2. Similarity of prioritized gene orthologs between humans and zebrafish.

Human gene	Zebrafish ortholog	Ensembl (%)	GeneCards (%)
PILRB	*pilrb*	20.26	-
ABCA7	*abca7*	26.10	48
TMEM199	*tmem199*	46.15	59.42
RDH5	*rdh5*	49.71	57.62
MMP9	*mmp9*	57.14	60.77
SARM1	*sarm1*	60.77	64.53
CNN2	*cnn2*	67.58	68.28
BLOC1S1	*bloc1s1*	81.70	90
C17orf90	No ortholog identified	-	-

Table S3. Primer sequences used for RT–qPCR analysis of key photoreceptor and RPE genes.

Target gene	Forward Primer (5'-3')	Reverse Primer (5'-3')
opn1lw2 (red opsin)	CCAACAGCAATAACACAAGGG	GCGACAACCACAAAGAACATC
opn1mw1 (green opsin)	GGCTGTGTAATGGAGGGATTC	ATGGTTTGCGGAGAATTTGAAG
opn1sw2 (blue opsin)	GGTTCCTTTCAGCACCATTG	AGAAGCCGAACACCATTACC
opn1sw1 (UV opsin)	TCATTTTCTCCTACTCACAGCTC	CACAAAAGAGCCAACCATCAC
Rho (rhodopsin)	AGTCCTGCCCAGACATCTAG	GTACTGTGGGTATTCGTATGGG
rpe65a	AGAGACGGGACGGTCTACAA	CCGTCATCCCAAAACTGTGC

Table S4. Summary of *Cnn2* expression across major cell classes in the mouse retinal single-cell RNA-seq dataset.

Cell class	Cell type full name	Mean raw UMI count per cell	*Cnn2*-expressing cells (%)
Endothelial	Endothelial Cells	1.448	57.80
Pericyte	Pericytes	0.580	36.83
RPE	Retinal Pigment Epithelium	0.207	15.40
Astrocyte	Astrocytes	0.121	9.46
Microglia	Microglia	0.095	8.39
MG	Müller Glia	0.049	3.55
RGC	Retinal Ganglion Cells	0.004	0.37
AC	Amacrine Cells	0.001	0.10
Rod	Rod Photoreceptors	0.001	0.08
BC	Bipolar Cells	0.000	0.05
Cone	Cone Photoreceptors	0.000	0.00
HC	Horizontal Cells	0.000	0.00

Table S5. Estimated retinal cell-type proportions from bulk RNA-seq deconvolution and comparisons between *Cnn2*-knockout and control samples.

Cell type	*Cnn2* KO 1	*Cnn2* KO 2	*Cnn2* KO 3	Control 1	Control 2	Control 3	Dirichlet test P value
AC	0.0000	0.0000	0.0000	0.0000	0.0000	0.0000	7.59E-1
BC	0.0000	0.0000	0.0000	0.0000	0.0000	0.0000	7.59E-1
Cone	0.0009	0.0003	0.0000	0.0000	0.0000	0.0000	4.78E-1
Endothelial	0.0006	0.0008	0.0091	0.0071	0.0066	0.0074	3.52E-6
HC	0.0010	0.0005	0.0000	0.0000	0.0008	0.0008	3.84E-1
MG	0.0292	0.0423	0.0249	0.0244	0.0266	0.0288	3.98E-3
Microglia	0.0000	0.0000	0.0000	0.0000	0.0000	0.0000	7.59E-1
Pericyte	0.0000	0.0000	0.0045	0.0044	0.0019	0.0016	2.37E-7
RGC	0.0225	0.0250	0.0181	0.0150	0.0119	0.0123	3.30E-2
RPE	0.0006	0.0004	0.0008	0.0008	0.0000	0.0000	1.96E-1
Rod	0.9453	0.9308	0.9426	0.9483	0.9522	0.9492	7.44E-4

Dataset S1 (separate file). SMR-based prioritization of candidate genes for AMD and cross-tissue replication of the prioritized genes. This dataset contains two worksheets. Worksheet 1 presents the summary-data-based Mendelian randomization (SMR) results obtained by integrating AMD GWAS summary statistics with whole-blood expression quantitative trait locus (eQTL) data, which were used to prioritize candidate genes for AMD. Worksheet 2 presents SMR replication results for the nine prioritized genes across multiple independent tissues. These analyses provide evidence of genetically predicted gene-expression associations with AMD but do not, by themselves, establish causality.

Dataset S2 (separate file). CARMA-based statistical fine-mapping of AMD-associated loci. This dataset reports CARMA fine-mapping results for AMD-associated loci, including variant-level association statistics, posterior inclusion probabilities (PIPs), and credible-set membership. These results were used to prioritize candidate causal variants for subsequent regulatory annotation and interpretation.

Dataset S3 (separate file). Differential gene expression and KEGG pathway enrichment analyses of *Cnn2*-knockout mouse retinas. This dataset contains two worksheets. Worksheet 1 presents the genome-wide differential expression results comparing retinas from Cnn2-knockout and control mice, including gene-level effect estimates and statistical significance measures. Worksheet 2 presents gene set enrichment analysis (GSEA) results for Kyoto Encyclopedia of Genes and Genomes (KEGG) pathways.

SI Reference

1. F.-F. Cheng *et al.*, Towards the identification of causal genes for age-related macular degeneration. *bioRxiv* 10.1101/778613, 778613 (2019).
2. B. R. Gorman *et al.*, Genome-wide association analyses identify distinct genetic architectures for age-related macular degeneration across ancestries. *Nat Genet* **56**, 2659-2671 (2024).
3. U. Võsa *et al.*, Large-scale cis- and trans-eQTL analyses identify thousands of genetic loci and polygenic scores that regulate blood gene expression. *Nature Genetics* **53**, 1300-1310 (2021).
4. L. G. Fritsche *et al.*, A large genome-wide association study of age-related macular degeneration highlights contributions of rare and common variants. *Nat Genet* **48**, 134-143 (2016).
5. C. Bycroft *et al.*, The UK Biobank resource with deep phenotyping and genomic data. *Nature* **562**, 203-209 (2018).
6. Z. Zhu *et al.*, Causal associations between risk factors and common diseases inferred from GWAS summary data. *Nature Communications* **9**, 224 (2018).
7. Y. Banda *et al.*, Characterizing Race/Ethnicity and Genetic Ancestry for 100,000 Subjects in the Genetic Epidemiology Research on Adult Health and Aging (GERA) Cohort. *Genetics* **200**, 1285-1295 (2015).
8. M. I. Kurki *et al.*, FinnGen provides genetic insights from a well-phenotyped isolated population. *Nature* **613**, 508-518 (2023).
9. J. M. Gaziano *et al.*, Million Veteran Program: A mega-biobank to study genetic influences on health and disease. *Journal of Clinical Epidemiology* **70**, 214-223 (2016).
10. C. A. De Leeuw, J. M. Mooij, T. Heskes, D. Posthuma, MAGMA: generalized gene-set analysis of GWAS data. *PLoS computational biology* **11**, e1004219 (2015).
11. A. Li *et al.*, mBAT-combo: a more powerful test to detect gene-trait associations from GWAS data. *The American Journal of Human Genetics* **110**, 30-43 (2023).
12. J. Yang, S. H. Lee, M. E. Goddard, P. M. Visscher, GCTA: a tool for genome-wide complex trait analysis. *American journal of human genetics* **88**, 76-82 (2011).
13. Z. Yang *et al.*, CARMA is a new Bayesian model for fine-mapping in genome-wide association meta-analyses. *Nature Genetics* **55**, 1057-1065 (2023).
14. C. P. Fulco *et al.*, Activity-by-contact model of enhancer–promoter regulation from thousands of CRISPR perturbations. *Nature Genetics* **51**, 1664-1669 (2019).
15. C. A. Boix, B. T. James, Y. P. Park, W. Meuleman, M. Kellis, Regulatory genomic circuitry of human disease loci by integrative epigenomics. *Nature* **590**, 300-307 (2021).
16. A. Kundaje *et al.*, Integrative analysis of 111 reference human epigenomes. *Nature* **518**, 317-330 (2015).
17. W. E. Kaminski *et al.*, Identification of a Novel Human Sterol-Sensitive ATP-Binding Cassette Transporter (ABCA7). *Biochemical and Biophysical Research Communications* **273**, 532-538 (2000).
18. W. S. Kim, G. J. Guillemin, E. N. Glaros, C. K. Lim, B. Garner, Quantitation of ATP-binding cassette subfamily-A transporter gene expression in primary human brain cells. *Neuroreport* **17**, 891-896 (2006).
19. S. L. Rosenthal, M. I. Kamboh, Late-Onset Alzheimer's Disease Genes and the Potentially Implicated Pathways. *Current genetic medicine reports* **2**, 85-101 (2014).
20. W. S. Kim *et al.*, Abca7 null mice retain normal macrophage phosphatidylcholine and cholesterol efflux activity despite alterations in adipose mass and serum cholesterol levels. *The Journal of biological chemistry* **280**, 3989-3995 (2005).

21. W. Li *et al.*, Hermansky-Pudlak syndrome type 7 (HPS-7) results from mutant dysbindin, a member of the biogenesis of lysosome-related organelles complex 1 (BLOC-1). *Nature genetics* **35**, 84-89 (2003).
22. R. Liu, J. P. Jin, Deletion of calponin 2 in macrophages alters cytoskeleton-based functions and attenuates the development of atherosclerosis. *J Mol Cell Cardiol* **99**, 87-99 (2016).
23. J. Tang *et al.*, A critical role for calponin 2 in vascular development. *The Journal of biological chemistry* **281**, 6664-6672 (2006).
24. C. M. Karch, L. A. Ezerskiy, S. Bertelsen, A. M. Goate, Alzheimer's Disease Risk Polymorphisms Regulate Gene Expression in the ZCWPW1 and the CELF1 Loci. *PloS one* **11**, e0148717 (2016).
25. A. Das *et al.*, Human diabetic neovascular membranes contain high levels of urokinase and metalloproteinase enzymes. *Investigative ophthalmology & visual science* **40**, 809-813 (1999).
26. H. E. Kaufman, The practical detection of mmp-9 diagnoses ocular surface disease and may help prevent its complications. *Cornea* **32**, 211-216 (2013).
27. B. L. Gruber *et al.*, Markedly elevated serum MMP-9 (gelatinase B) levels in rheumatoid arthritis: a potentially useful laboratory marker. *Clinical immunology and immunopathology* **78**, 161-171 (1996).
28. S. Hiratsuka *et al.*, MMP9 induction by vascular endothelial growth factor receptor-1 is involved in lung-specific metastasis. *Cancer cell* **2**, 289-300 (2002).
29. N. Sakalihasan, P. Delvenne, B. V. Nusgens, R. Limet, C. M. Lapiere, Activated forms of MMP2 and MMP9 in abdominal aortic aneurysms. *Journal of vascular surgery* **24**, 127-133 (1996).
30. K. J. Ryan *et al.*, A human microglia-like cellular model for assessing the effects of neurodegenerative disease gene variants. *Science translational medicine* **9** (2017).
31. Y. I. Li, G. Wong, J. Humphrey, T. Raj, Prioritizing Parkinson's disease genes using population-scale transcriptomic data. *Nature communications* **10**, 994 (2019).
32. M. Nakamura, Y. Hotta, A. Tanikawa, H. Terasaki, Y. Miyake, A high association with cone dystrophy in Fundus albipunctatus caused by mutations of the RDH5 gene. *Investigative ophthalmology & visual science* **41**, 3925-3932 (2000).
33. Y. Makiyama *et al.*, Cone abnormalities in fundus albipunctatus associated with RDH5 mutations assessed using adaptive optics scanning laser ophthalmoscopy. *American journal of ophthalmology* **157**, 558-570.e551-554 (2014).
34. C. W. Lin, C. Y. Chen, S. J. Cheng, H. T. Hu, Y. P. Hsueh, Sarm1 deficiency impairs synaptic function and leads to behavioral deficits, which can be ameliorated by an mGluR allosteric modulator. *Frontiers in cellular neuroscience* **8**, 87 (2014).
35. C. W. Lin, Y. P. Hsueh, Sarm1, a neuronal inflammatory regulator, controls social interaction, associative memory and cognitive flexibility in mice. *Brain, behavior, and immunity* **37**, 142-151 (2014).
36. J. Yang *et al.*, Pathological axonal death through a MAPK cascade that triggers a local energy deficit. *Cell* **160**, 161-176 (2015).
37. J. M. Osterloh *et al.*, dSarm/Sarm1 is required for activation of an injury-induced axon death pathway. *Science (New York, N.Y.)* **337**, 481-484 (2012).
38. J. C. Jansen *et al.*, TMEM199 Deficiency Is a Disorder of Golgi Homeostasis Characterized by Elevated Aminotransferases, Alkaline Phosphatase, and Cholesterol and Abnormal Glycosylation. *American journal of human genetics* **98**, 322-330 (2016).
39. A. L. Miles, S. P. Burr, G. L. Grice, J. A. Nathan, The vacuolar-ATPase complex and assembly factors, TMEM199 and CCDC115, control HIF1alpha prolyl hydroxylation by regulating cellular iron levels. *eLife* **6** (2017).
